# Decoding Myosin-3 mutational hotspots: Linking deleterious variants to Duchenne muscular dystrophy severity and psychiatric comorbidities

**DOI:** 10.1371/journal.pone.0328503

**Published:** 2025-07-15

**Authors:** Mohammed Ageeli Hakami, Ahad Amer Alsaiari, Taj Mohammad, Anas Shamsi

**Affiliations:** 1 Department of Clinical Laboratory Sciences, College of Applied Medical Sciences, Shaqra University, Al- Quwayiyah 19257, Riyadh, Saudi Arabia; 2 King Salman Center for Disability Research, Riyadh 11614, Saudi Arabia; 3 Department of Clinical Laboratory Sciences, College of Applied Medical Sciences, Taif University, Taif 21944, Saudi Arabia; 4 Centre for Interdisciplinary Research in Basic Sciences, Jamia Millia Islamia, New Delhi 110025, India; 5 Centre of Medical and Bio-Allied Health Sciences Research, Ajman University, Ajman, United Arab Emirates; Amity University, INDIA

## Abstract

Duchenne muscular dystrophy (DMD) is a severe neuromuscular disorder primarily caused by mutations in the dystrophin gene, leading to progressive muscle degeneration. While the loss of dystrophin is central to DMD pathogenesis, impaired muscle regeneration further exacerbates disease severity. As *MYH3*-encoding Myosin-3 is involved in muscle development and regeneration, we examined how it could be added to the list of possible contributors to DMD pathology. This study employed various computational tools such as PolyPhen-2, SIFT, and I-Mutant to analyze 486 *MYH3* missense mutations and predict the structural and functional implications. We discovered 89 deleterious substitutions, of which 80 were pathogenic. Of these, 45 mutations were identified as likely to pathogenically alter Myosin-3 solubility, and 5 (G182A, R244C, R244H, H285Y, N483S) fell within evolutionarily conserved regions. The mutant G182A is of particular interest as it lies within the ATP-binding site, which may lead to an impairment of energy-dependent myosin activity. These mutations likely impair muscle regeneration, potentially intensifying the severity of dystrophy. Furthermore, we hypothesize that these functional deficiencies may not be limited to muscle pathogenesis and could be related to the development of neuropsychiatric comorbidities observed in DMD, although this remains to be experimentally confirmed. Our results emphasize the relevance of Myosin-3 in the pathogenesis of DMD and the importance of combined research on neuromuscular and psychiatric aspects to improve therapeutic approaches.

## 1. Introduction

Duchenne muscular dystrophy (DMD) is a devastating X-linked recessive neuromuscular disorder with an incidence of 1 per 5000 male live births worldwide [1]. It is the most common inherited neuromuscular disorder and occurs without race or ethnic predilection [2]. DMD primarily arises from mutations in the DMD gene (OMIM 300377, Xp21.2-p21.1), which encodes dystrophin, a critical structural protein in muscle cells [3]. Being one of the largest known human genes, the *DMD* gene is especially prone to mutations, particularly deletions, which account for the majority of DMD cases [4]. The clinical presentation of DMD is one of muscle wasting and loss of strength, resulting in severe disability and premature death, usually from respiratory or cardiomyopathy [5]. Aside from these devastating effects on muscle structure, new insight from research indicates that individuals with DMD are still more likely to develop psychiatric-related issues, which include anxiety, depression, and difficulties in thinking, as they combat muscle atrophy [6,7]. Psychiatric comorbidities in DMD, such as anxiety and depression, likely arise from a combination of dystrophin loss in the CNS, secondary psychosocial stress due to progressive disability, and shared molecular pathways affecting cytoskeletal regulation [8]. Understanding these relationships is essential for a comprehensive interpretation of DMD pathobiology.

Dystrophin is a cytoplasmic protein that is essential in bridging the cytoskeleton and the extracellular matrix via the dystrophin-associated protein complex (DAPC) [9]. The mechanical connection is important to preserve the structural stability of the muscle fibres, especially since the muscle tissues undergo repeated cycles of contraction and relaxation [10]. In the context of DMD, when dystrophin is not functional, CK8 maintains the sarcolemma exposed to damage, and chronic inflammation and cycles of necrosis and regeneration are established, leading to a series of pathological events [11]. These modifications eventually lead to the replacement of muscle fibres by adipose and connective tissue, which manifests as a decrease in muscle mass and function. In the mild form of the disease, Becker’s muscular dystrophy (BMD), they share the pathological features similar to DMD, but are less severe due to partial reduction in dystrophin [12]. This underscores the threshold level of dystrophin required for normal muscle function and the disastrous consequences of its absence.

Although the primary consequences of dystrophin deficiency are well documented in DMD pathogenesis, the secondary effects on other muscle proteins are less well understood [13]. One of these proteins, Myosin-3, is produced from the *MYH3* gene. Myosin-3 belongs to the myosin heavy chain family and participates in muscle contraction [14]. It also has an important role during muscle development and repair, which is very important in a situation of continuous muscle damage and regeneration, as it occurs in DMD [15,16]. While not directly implicated in early DMD pathogenesis, Myosin-3 serves as a key marker of muscle regeneration and injury [13]. The presence of developmental myosins such as Myosin-3 in muscle fibers is indicative of active muscle regeneration, a compensatory mechanism in response to the continuous muscle damage occurring in DMD [17]. Therefore, Myosin-3 can serve as a valuable biomarker for assessing disease progression and the effectiveness of therapeutic interventions in DMD [13].

In the pathological context of DMD, muscle fibers are subjected to constant cycles of damage and repair [18]. Regeneration of muscle is an important determinant in disease progression [19]. Proteins involved in muscle regeneration, such as Myosin-3, are upregulated during these regenerative processes [13,20]. However, mutations in the gene encoding the Myosin-3 protein can have substantial consequences on its function and expression, which influences muscle regeneration [21,22]. These mutations can disturb the protein structure of Myosin-3 protein and consequently hinder it from effectively participating in muscle fiber generation and repair. This would further deteriorate the muscle trauma observed in DMD and accelerate the disease pathology. Studies suggest that Myosin-3 is not strictly muscle-specific. Expression profiling and immunohistochemistry have detected traces of developmental myosin heavy chains in certain brain regions, including the hippocampus and cerebellum. Functionally, Myosin-3 participates in actin cytoskeletal dynamics that are conserved between muscle and neuronal cells, which could underlie both muscle regeneration deficits and neuropsychiatric comorbidities in DMD. These observations warrant further studies to dissect Myosin-3’s dual roles in muscle and CNS physiology.

Recent progress in genomic technologies has led to the identification and characterization of several mutations in the *MYH3* gene [21,23]. This has added to our knowledge of the functional consequences of those mutations. We investigated the impacts of mutations on the Myosin-3 protein through state-of-the-art sequence and structural analysis methods. ATP-binding site of Myosin-3 is key to its motor function, which mediates the hydrolysis of ATP to form the mechanical force as required by muscle contraction [24]. Mutations in this position can interfere with ATP binding and/or ATP hydrolysis and thus have the potential to affect Myosin-3 motor function. Such alteration can profoundly affect the function and regeneration of muscle fibers, worsening the muscle pathology observed in DMD. Likewise, other reported mutations located in conserved regions of Myosin-3 might have an impact in terms of structural stability and association with other proteins that participate in muscle contraction and repair. High-confidence pathogenic mutations are valuable in support and explanation of the molecular etiology of DMD, but also for identifying new avenues to therapeutic intervention.

## 2. Materials and methods

### 2.1 Retrieval of data

The protein sequence of human Myosin-3 was taken from the UniProt database (UniProt ID: P11055) in FASTA format. A table of single-point mutations was prepared from the data available in dbSNP [25] and Ensembl [26] databases. In addition, for each of the 486 variants, we retrieved minor allele frequencies (MAFs) from gnomAD v3.1 (https://gnomad.broadinstitute.org/) and dbSNP (https://www.ncbi.nlm.nih.gov/snp/) to report their prevalence in the general population and, where available, in-patient cohorts. Variants were filtered by MAF < 1% to focus on rare substitutions potentially relevant to DMD pathology. Duplicate mutations were eliminated to avoid redundancy. The three-dimensional structure of the Myosin-3 protein was downloaded from AlphaFoldDB (AlphaFold ID: AF-P11055-F1) [27]. We have extracted the structure of the Myosin motor domain of Myosin-3, which contains D86 to D779 amino acid residues. So, we have taken only those mutations that lie in that region.

### 2.2 Identification of damaging/deleterious mutations

Four web-based predictors were utilized for the sequence-based prediction of damaging/deleterious single amino acid substitutions. These tools use the FASTA sequence or UniProt ID along with the mutations as input and return the deleterious and benign mutations. SIFT [28,29], PolyPhen2 [30], Mutation Assessor [31], and FATHMM [32] are the web-based state-of-the-art tools used for this analysis. These tools evaluate the comparative and physicochemical properties of amino acid substitutions to estimate their potentially damaging effects on protein structure and function. PolyPhen-2 provides the Position-Specific Independent Count (PSIC) score; a PSIC score greater than 0.09 indicates a deleterious mutation. Based on its inference model, FATHMM classifies single amino acid substitutions as either tolerated or damaging. A SIFT score of 0.05 or less signifies an intolerable mutation. The Mutation Assessor yields an FI score for each variant, with scores above 2.00 indicating a deleterious variant.

### 2.3 Characterization of protein stability changed by mutations

Four different web-based predictors were used for structure-based prediction which are mCSM [33], PremPS [34], MAESTROweb [35], and DynaMut2 [36]. All these tools utilize the PDB structure file of the wild-type protein as input and use the atomic coordinates to determine the stability of variants by calculating the folding free energy. They assess the change in protein stability between the wild-type and mutant proteins. Structure-based tools evaluate the impact of a point mutation on protein stability by calculating the Gibbs free energy change (ΔΔ*G*). For a given single-point mutation, mCSM, PremPS, and DynaMut2 predict it as a destabilizing mutation if the ΔΔ*G* is greater than 0. Conversely, MAESTROweb considers a mutation to alter protein stability if the ΔΔ*G* is less than 0.

### 2.4 Analysis of mutation-induced pathogenicity

Pathogenicity predictors evaluate the disease phenotype association of a mutation in a protein. PhD-SNP [37], SNPs&GO [38] and MutPred2 [39] are web-based tools used for pathogenicity prediction. Disease phenotype prediction tools take the FASTA sequence file and mutations as input. PhD-SNP and SNPs&GO use SVM-based classifiers to classify the disease-associated variants [40]. MutPred2 employs neural network-based classifiers, utilizing predicted physicochemical properties and sequence conservation as primary features. A MutPred2 score greater than 0.05 indicates a pathogenic single-point mutation.

### 2.5 Analysis of aggregation propensity

The Solubility based on Disorder and Aggregation (SODA) tool (http://protein.bio.unipd.it/soda/) can determine the tendency for aggregation, disorder, helix, and strand formation resulting from mutations [41]. This tool accepts protein sequences or PDB format structure files as input. Using the PASTA 2.0, ESpritz-NMR, and Fells algorithms, SODA predicts the effects of mutations such as insertion, deletion, substitution, and duplication. It assigns a final score based on the solubility variation between the wild-type and mutant proteins [42].

### 2.6 Analysis of conserved residues

Understanding evolution and the structure and function of a protein depends on the conservation of amino acids. ConSurf (https://consurf.tau.ac.il/) and AmoAI (https://kornmann.bioch.ox.ac.uk/amo/) tools were exploited to assess the conservation level of residues at specific positions by using multiple sequence alignment [43]. The ConSurf tool determines each residue’s conservation degree using the maximum likelihood (ML) or empirical Bayesian method. There are three possible ConSurf scores: 1 for the least conserved sites, 5 for intermediately conserved positions, and 9 for highly conserved positions. The pre-estimated scores for well-known PDB structures are in the ConSurf-DB.

### 2.7 Residual frustration analysis

The investigation of residual frustration analysis of proteins provides useful information about the levels of structural frustration within a protein [44,45]. To analyze the residual frustration on Myosin-3, we used the Frustratometer server (http://frustratome-ter.qb.fcen.uba.ar/). This module allowed us to obtain the per-individual and group residual indices for the Myosin-3 structure. Inspired by the Energy Landscape Theory, the Frustratometer is an algorithm that attempts to determine to what extent a protein molecule is locally frustrated [46]. This concept gives insights into the biological rationale of proteins by investigating how energy flows through their structures and how mutations can change these flows. Some high local frustration clusters reveal biologically interesting regions, for instance, binding sites, whereas the least frustrated contacts compose the folding core of the molecule. It can also be used to simulate the effects of electrostatics on the local frustration values.

### 2.8 Analysis of protein-protein interaction

**Fig 1:**
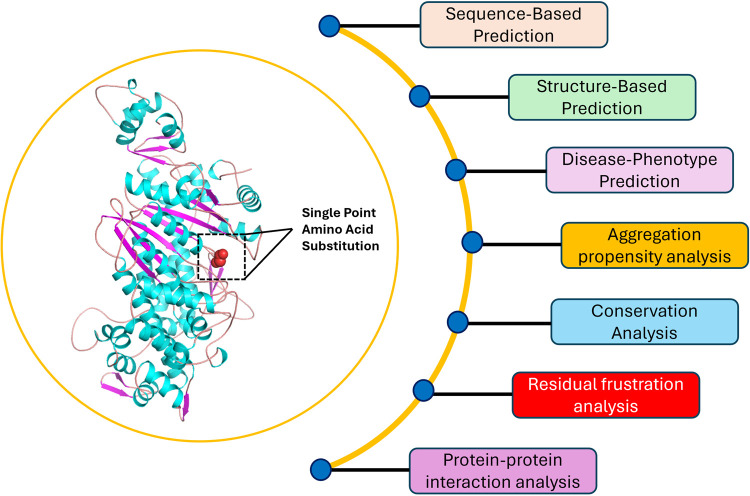
Visualization depicting the workflow pipeline utilized in this study. The left panel shows the domain architecture of Myosin-3 colored by secondary structure elements. The right panel depicts the workflow schematic of the different approaches used in the study.

Analyzing protein-protein interactions is crucial for understanding cellular mechanisms and the interplay between various proteins, especially when investigating the impact of abnormal proteins and their links to diseases [47,48]. The STRING database was used to analyse the interaction of Myosin-3 [49]. Here, overlaid confidence score of 0.700 was used to generate interaction networks for Myosin-3. In order to better define these interactions, we retrieved the 3D structures of these interacting proteins using SWISS-Model (https://swissmodel.expasy.org). Fig 1 illustrates the overall methodology used in this study.

## 3. Results and discussion

### 3.1 Identification of damaging/deleterious mutations

**Fig 2:**
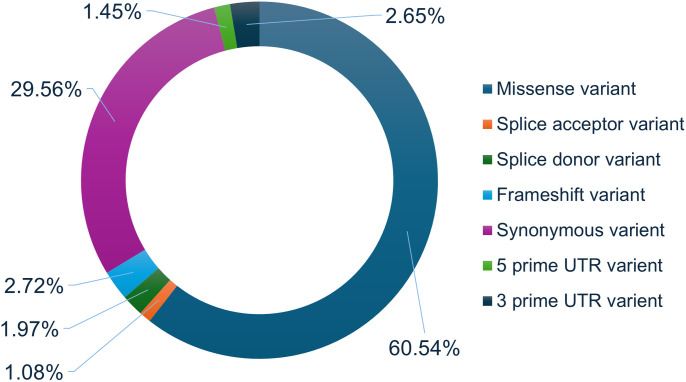
Depiction of the SNPs found within the MYH3 gene utilizing the dbSNP database.

A total of 486 single amino acid substitutions in Myosin-3 were collected from the dbSNP (http://www.ncbi.nlm.nih.gov/snp) and Ensembl (http://www.ensembl.org/) databases (Fig 2). A multi-tier approach was employed to identify damaging and destabilizing mutations in the Myosin-3 protein. High-confidence mutations were obtained through sequence and structure-based web platforms. Multiple tools were utilized to mitigate false results and ensure accuracy. The sequence-based analysis involved four web-based tools: SIFT, PolyPhen2, Mutation Assessor, and FATHMM (Table S1 in S1 File). For the structure-based approach, mCSM, PremPS, MAESTROweb, and DynaMut2 were utilized to analyze single amino acid substitutions of Myosin-3 protein (Table S2 in S1 File). Only high-confidence variants underwent further investigation, including pathogenicity prediction tools such as PhD-SNP, SNPs&GO, and MutPred2 (Table S3 in S1 File).

### 3.2 Characterization of protein stability

Single-point mutations associated with disease can significantly impact protein stability [50]. The Gibbs free energy change (ΔΔ*G*) is a crucial metric for assessing the mutational impact on protein stability. ΔΔ*G* is calculated as Δ*G* = *G*u - *G*f, where *G*f represents the energy of the folded protein and *G*u denotes the energy of the unfolded protein. The change in protein stability and free energy landscape is then determined as ΔΔ*G* = *G*m − *G*w, with *G*m representing the mutant protein and *G*w the wild-type protein. A negative ΔΔ*G* value suggests a more stabilizing mutation, whereas a positive value indicates destabilizing mutations. In our investigation, we employed four different structure-based stability predictors: SDM, MAESTROweb, PremPS, and CUPSAT. These tools typically adopt a neural network-based approach that integrates various biophysics-based theories.

**Fig 3:**
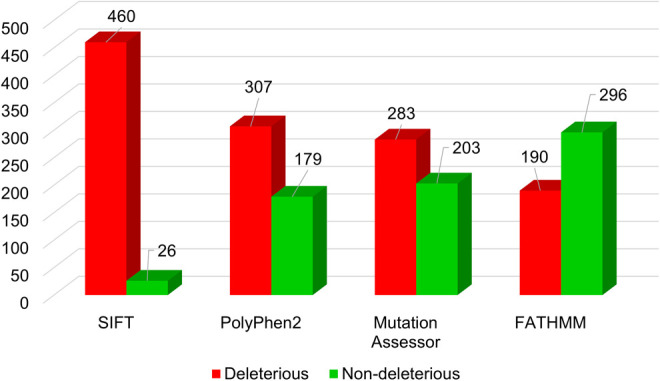
Sequence-based prediction of deleterious mutations in Myosin-3. The bar plots showed predictions from SIFT, PolyPhen-2, Mutation Assessor, and FATHMM. Red and green showed deleterious and non-deleterious mutations, respectively.

**Fig 4:**
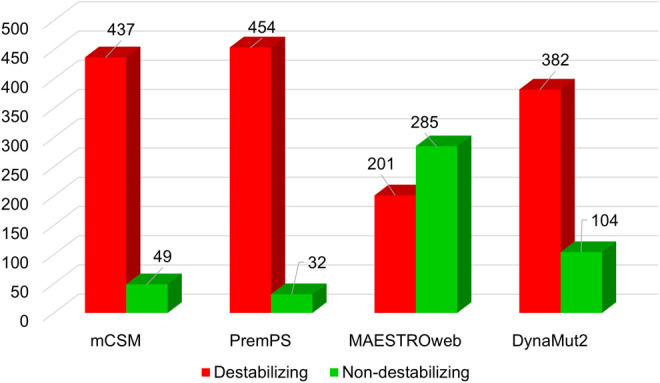
Structure-based prediction of destabilizing mutations in Myosin-3. The bar plots showed predictions from mCSM, PremPS, MAESTROweb, and DynaMut2. Red and green showed destabilizing and non-destabilizing mutations, respectively.

A detailed analysis was conducted on 486 single amino acid substitutions within the Myosin-3 myosin motor domain, utilizing a sequence-based approach. SIFT, PolyPhen2, Mutation Assessor, and FATHMM predicted 460, 307, 283, and 190 mutations as deleterious, respectively (Fig 3). Concurrently, structure-based predictions by mCSM, PremPS, MAESTROweb, and DynaMut2 identified 437, 454, 201, and 382 variations as destabilizing mutations (Fig 4). To refine our analysis, we focused on high-confidence mutations, selecting those predicted to be deleterious or destabilizing by all tools in both approaches. This rigorous criterion resulted in a subset of 89 mutations. These mutations, consistently predicted to be deleterious by sequence-based tools and destabilizing by structure-based tools, were subjected to further analysis to determine their potential association with disease phenotypes or pathogenicity. The concordance among multiple predictive tools reinforces the reliability of these high-confidence mutations.

### 3.3 Analysis of mutation-induced pathogenicity

**Fig 5:**
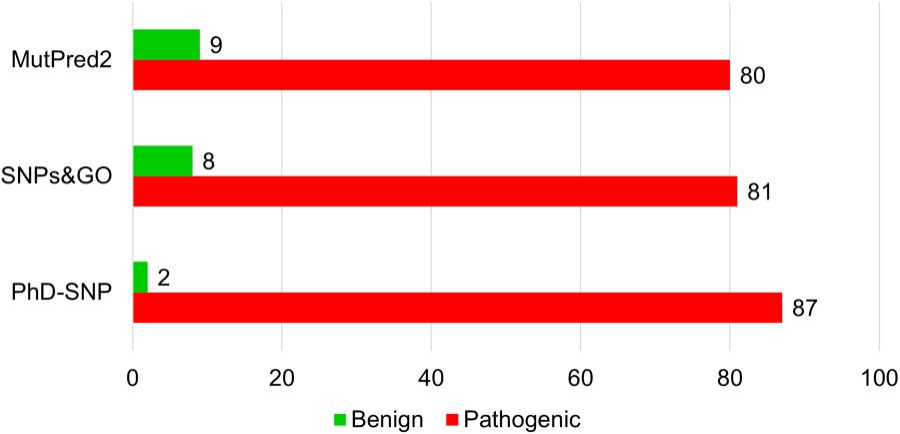
Structure-based prediction of pathogenic mutations in Myosin-3. The bar plots showed predictions from PhD-SNP, SNPs&GO, and MutPred2. Red and green showed pathogenic and benign mutations, respectively.

Disease phenotype association prediction was conducted utilizing PhD-SNP, SNPs&GO, and MutPred2 web tools. These tools categorize mutations as pathogenic or benign based on their pathogenicity scores. From the pool of 89 high-confidence variations obtained from sequence and structure-based analysis, PhD-SNP, SNPs&GO, and MutPred2 predicted 87, 81, and 80 mutations as disease-associated, respectively (Fig 5). Among these, 80 high-confidence mutations were identified as pathogenic by all disease phenotype prediction tools. The high overlap of 80 pathogenic mutations identified by all three tools provides a set of high-confidence variants likely to be implicated in disease phenotypes. This agreement enhances the validity of these mutations as targets for further functional studies and potential therapeutic interventions. Moreover, the discrepancies observed for the remaining mutations suggest areas for future refinement in prediction algorithms and underline the importance of using multiple tools for comprehensive pathogenicity assessments.

### 3.4 Analysis of aggregation propensity

The function of a protein can be significantly impacted by its solubility [51,52]. Insoluble regions of a protein tend to aggregate, potentially leading to various complex diseases such as Alzheimer’s [53], amyloidosis [54], Parkinson’s disease [54], and DMD [55,56]. The average solubility of protein point mutations was predicted using the SODA (Solubility based on Disorder and Aggregation) approach. This approach calculates the overall disorder, helix, and strand propensities that arise from these mutations. Of the 80 variants detected through disease phenotype prediction, 45 resulted in a reduction in the solubility of the protein through single amino acid substitutions (Table 1). Their prevalence suggests that altered solubility plays a significant role to the impaired function of the Myosin-3 motor domain. These data demonstrate the significance of solubility in the context of disease-associated mutations. Lower solubility caused by amino acid replacements, for example, can cause aggregation of the protein itself, which is characteristic of various neurodegenerative and muscular disorders, such as DMD. Identification of these 45 mutations gives insight into the molecular pathogenesis possibly causing disease phenotypes linked to Myosin-3.

**Table 1:** Predicted aggregated mutants of the Myosin-3 protein using the SODA server.

S. No.	Mutation	Helix	Strand	Aggregation	Disorder	SODA Score	Soda Prediction
1.	N105S	−0.597	0.708	−6.305	0.003	−6.147	Less soluble
2.	L106V	−2.785	2.546	−36.719	0.003	−37.203	Less soluble
3.	R109H	0.462	−0.394	−13.829	−0.009	−13.746	Less soluble
4.	P153S	−0.003	0.686	−4.455	0.099	−2.911	Less soluble
5.	H154Y	−1.064	1.388	−4.179	−0.42	−4.419	Less soluble
6.	M166V	−4.382	3.888	−11.65	0.057	−12.582	Less soluble
7.	I175F	−0.026	−0.035	−3.783	−0.136	−4.15	Less soluble
8.	G182A	0.175	−0.045	−1.034	−0.039	−0.909	Less soluble
9.	D219G	−8.398	6.887	−0.907	−0.026	−4.061	Less soluble
10.	R244C	−0.035	0.084	−5.286	−0.131	−5.63	Less soluble
11.	R244H	−0.054	−0.015	−0.204	−0.029	−0.433	Less soluble
12.	E281K	0.201	−0.198	−3.26	0.005	−3.233	Less soluble
13.	H285Y	−2.646	2.294	−53.827	0.042	−54.497	Less soluble
14.	F287V	−2.766	2.309	−25.684	0.023	−26.588	Less soluble
15.	Q289H	−2.719	2.182	−21.871	0.023	−22.906	Less soluble
16.	Y311C	−0.425	0.199	0.452	−0.014	−0.125	Less soluble
17.	A336T	−3.161	2.651	−3.285	−0.06	−4.361	Less soluble
18.	L400F	−0.737	0.526	−2.934	−0.017	−3.365	Less soluble
19.	Q420R	−0.411	0.23	−3.59	−0.005	−3.935	Less soluble
20.	L436F	−0.734	0.44	−31.689	0.006	−32.239	Less soluble
21.	R443C	−0.035	−0.152	−46.681	−0.018	−47.271	Less soluble
22.	D449V	−0.27	0.959	−31.701	−0.057	−30.635	Less soluble
23.	E467A	4.782	−2.939	−11.67	0.003	−8.116	Less soluble
24.	N483S	−0.32	0.204	−5.263	0	−5.564	Less soluble
25.	E501K	0.531	−0.335	−1.908	0.001	−1.504	Less soluble
26.	D546G	−2.006	1.411	−0.826	−0.056	−2.205	Less soluble
27.	A584E	−6.707	5.198	−0.236	0.078	−2.962	Less soluble
28.	W594C	−1.696	1.009	0	−0.005	−1.471	Less soluble
29.	L617P	−9.338	5.808	6.593	−0.13	−0.586	Less soluble
30.	L621F	−1.868	1.421	−3.713	−0.155	−4.891	Less soluble
31.	L621R	−6.243	4.347	2.268	−0.308	−1.926	Less soluble
32.	P676A	2.777	−1.037	−18.162	−0.053	−14.875	Less soluble
33.	N677S	−0.16	0.214	−2.501	−0.007	−2.477	Less soluble
34.	Y716C	−0.163	−0.024	−1.425	0.002	−1.893	Less soluble
35.	G769S	0.692	−0.263	−4.95	0.005	−4.072	Less soluble

### 3.5 Analysis of evolutionarily conserved residues

**Fig 6:**
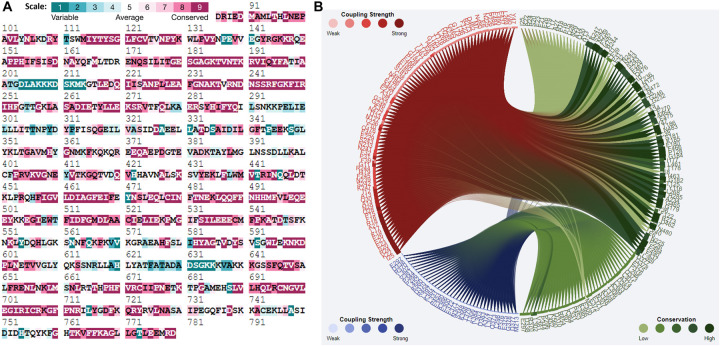
Conservation analysis. (A) ConSurf plot of evolutionarily conserved residues in Myosin-3. (B) Residual communities in green showing highly conserved residues in Myosin-3.

Evolutionary conservation analysis provides insights into residue functional importance and localized evolutionary constraints [57,58]. Conserved residues play a crucial role in maintaining the structural integrity of a protein [59]. The likelihood of an amino acid undergoing mutation is influenced by its level of conservation [60]. We used the ConSurf server to analyze the conservation of residues in the human Myosin-3 protein, specifically in the Myosin motor domain. The residue-specific APD index showed that the APF remarkably varied among amino acid residues, and the conservation analysis indicated that four sites (G182, R244, H285 and N483) were more conserved than other sites (Fig 6). This high degree of conservation suggests that these residues are essential for the function of the Myosin motor. As a good example, the amino acid G182A at the ATP binding pocket was the most conservative residue and had the highest possibility of being protein-aggregating. This supports the notion that mutations at G182 could critically impact on the protein stability and function that eventually may promote aggregate formation, commonly found in pathological conditions. In general, the phylogenetic conservation analysis points to the relevance of these (G182, R244, H285, and N483) residues on the Myosin-3 protein and contributes to understanding the specific roles in keeping the structural and active functions of the protein.

### 3.6 Analysis of residual frustration

**Fig 7:**
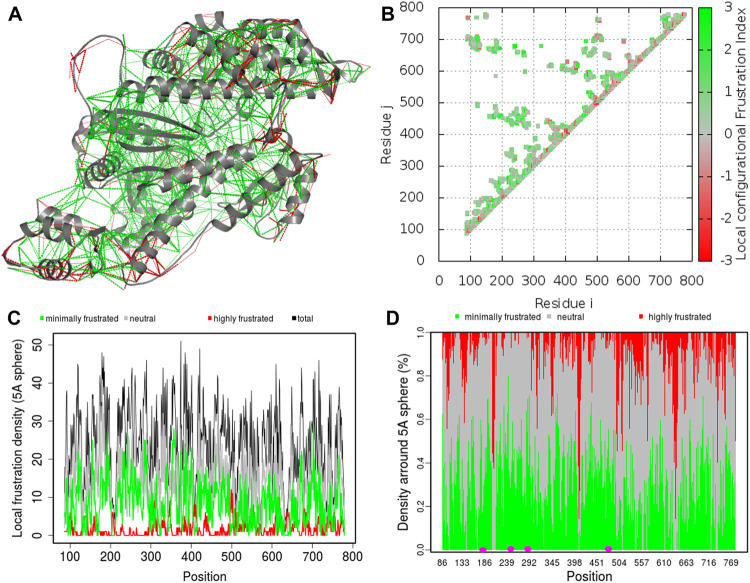
Residual frustration maps. (A) 3D structure of Myosin-3 with frustration index. (B) Residue-residue contact level in Myosin-3. (C) The frustration contact map in Myosin-3. (D) The pointed frustration contact map of Myosin-3 with mutation sites is highlighted in magenta.

Analysis of residual frustration offers valuable insights into the intricate energy landscapes of proteins and aids in understanding the interplay between protein structure, stability, and function [61]. By examining frustration within protein structures, specific sites of frustration can be identified [62]. We then investigated the local frustration in Myosin-3. With frustration indices, we measured the stability of native contacts as compared with all the possible contacts at the degree of frustration. Our analysis revealed varying levels of local frustration across the Myosin-3 structure (Fig 7). In addition, we also studied the contents of the configurational frustration at the residue-residue contact level and found consistent frustration patterns in the contact map. The lattice showed marginal frustration at some points. Contacts with mutated contacts G182A, R244C, R244H, H285Y, and N483S exhibited low frustration. Mutations of such residues in Myosin-3 might perturb stability and hence function of the protein, and may play a role in DMD pathogenesis.

### 3.7 Protein-protein interaction and functional characterization

**Fig 8:**
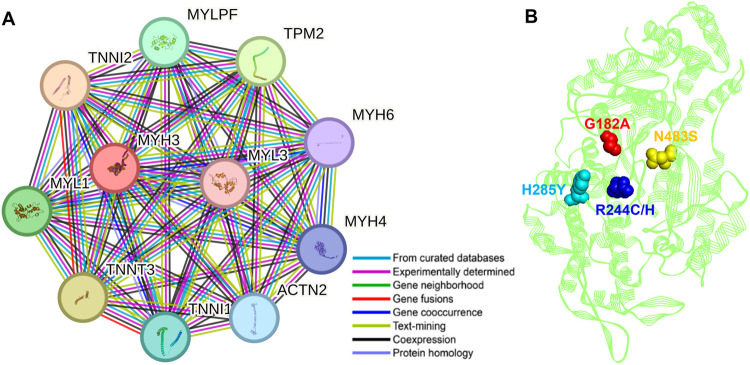
Protein-protein interaction and mutational landscape of Myosin-3. (A) Analysis of the Myosin-3 protein-protein interaction network reveals its interacting partners. The network was generated by STRING at a confidence level of 0.700. (B) Mutational positions on the Myosin-3 structure.

To understand the regulations of the Abn-Myosin-3 protein, it is critical to analyze its interaction partners. Several strongly interconnected proteins were recognized from the STRING database for protein-protein interaction network, (Fig 8). The Myosin-3 also interacted with the MYH4, MYH6, MYL1, MYL3, and MYLPF, which puts it into an extended Myosin superfamily (Fig 8A). This superfamily is best known for its involvement in muscle movement and a variety of motility phenomena in eukaryotic cells. Myosin-3 was also shown to bind the contractile proteins, namely troponins TNNI1, TNNI2, and TNNT3, which, with tropomyosin, control the calcium sensitivity of the contractile apparatus of striated muscles, and this is a mechanism that is important for the fine-tuning of contraction and relaxation of the muscle *in vivo*.

Notably, Myosin-3 also interacts with TPM2 (β-Tropomyosin), whose function includes binding to actin filaments, both in muscle and smooth muscle. It is essential for the regulation of muscle contraction, together with the troponin complex, in a calcium-dependent manner. Mutations in myosins, including Myosin-3, are responsible for a range in inherited muscle diseases called myopathies. Our analysis uncovered 5 distinct missense mutations in the conserved Myosin-3 region, G182A, R244C, R244H, H285Y, and N483S (Fig 8B). These mutations were anticipated to be deleterious, destabilizing, and pathogenic. These mutations result in protein aggregation and a fall in minimally frustrated regions of the protein, which are suspected to be responsible for Myosin-3-related DMD pathogenesis.

Taking together, this study is based exclusively on *in silico* predictions and public database frequencies; structural ΔΔ*G* and pathogenicity scores may not fully capture context-dependent effects in muscle or neural tissues. Experimental validation, such as ATPase assays, solubility tests in cell lines, and patient‐derived myoblast studies, is required to confirm the predicted functional impacts and to translate these findings into clinical biomarkers or therapeutic targets.

## 4. Conclusions

This study explores the complex interactions between Myosin-3 genetic changes and the development of DMD pathology. Our integrated computational analysis combined multiple evaluation tools to discover 89 harmful mutations in Myosin-3, predicting 80 of them as disease-causing. The study focused on determining the effects of these Myosin-3 mutations on protein structure, function, and disease link. Analysis showed that 45 distinct mutations in Myosin-3 affect the protein by lowering its solubility. The loss of protein solubility may lead to harmful protein clumps that intensify muscle destruction in people with DMD. The study highlights that mutations in conserved regions of Myosin-3, specifically G182, R244, H285, and N483, may critically impair protein function and stability. Myosin-3 motor function relies on the G182A mutation at the ATP binding site, making this specific genetic variation important for understanding DMD development. Mutations occurring in minimally frustrated regions of Myosin-3 are likely to compromise its stability and function, promoting the development of DMD. The damaging mutations G182A, R244C, R244H, H285Y, and N483S in Myosin-3 block its protein interactions and might trigger DMD formation. Although our results identify high-confidence deleterious mutations, these computational predictions must be validated experimentally (e.g., *in vitro* ATPase assays, animal models) to confirm their pathogenic roles. Future studies need to exploit these in-silico findings to better understand the biological effects. Overall, the study suggests how Myosin-3 mutations affect DMD at both molecular and structural levels while suggesting new paths to study their connection to psychiatric symptoms. Finding the links between muscle disease and psychiatric conditions in DMD patients will lead to therapies that treat both symptoms together.

## Supporting information

S1 File Table S1. List of deleterious mutations in Myosin-3 predicted through sequence-based tools. Table S2. List of destabilizing mutations in Myosin-3 predicted through structure-based tools. Table S3. List of pathogenic mutations in Myosin-3 predicted through structure-based tools.
(DOCX)
