# Supplementary material for: Decoding Myosin-3 mutational hotspots: Linking deleterious variants to Duchenne muscular dystrophy severity and psychiatric comorbidities
Source: PLoS One. 2025 Jul 15;20(7):e0328503. doi: 10.1371/journal.pone.0328503 (PMC12262874; doi:10.1371/journal.pone.0328503)
Supplement: S1 File — Table S1. List of deleterious mutations in Myosin-3 predicted through sequence-based tools. Table S2. List of destabilizing mutations in Myosin-3 predicted through structure-based tools. Table S3. List of pathogenic mutations in Myosin-3 predicted through structure-based tools. (DOCX) [file pone.0328503.s001.docx]

*Supplementary data*

**Decoding Myosin-3 Mutational Hotspots: Linking Deleterious Variants to Duchenne Muscular Dystrophy Severity and Psychiatric Comorbidities**

Mohammed Ageeli Hakami^1,2,*^, Ahad Amer Alsaiari^3^, Taj Mohammad^4,#^, Anas Shamsi^5,#^

*^1^Department of Clinical Laboratory Sciences, College of Applied Medical Sciences, Shaqra University, Al- Quwayiyah-19257, Riyadh, Saudi Arabia*

*^2^King Salman Center for Disability Research, Riyadh 11614, Saudi Arabia*

*^3^Department of Clinical Laboratory Sciences, College of Applied Medical Sciences, Taif University, PO Box 11099, Taif 21944, Saudi Arabia.*

*^4^Centre for Interdisciplinary Research in Basic Sciences, Jamia Millia Islamia, New Delhi 110025, India.*

*^5^Centre of Medical and Bio-Allied Health Sciences Research, Ajman University, Ajman, United Arab Emirates.*

*^*^Corresponding Author:*

**Mohammed Ageeli Hakami, PhD**

E-mail: [m.hakami@su.edu.sa](mailto:m.hakami@su.edu.sa)

*^#^Co-Corresponding Authors:*

**Taj Mohammad, M.Phil., PhD**

E-mail: [taj144796@st.jmi.ac.in](mailto:taj144796@st.jmi.ac.in)

*^#^Co-Corresponding Authors:*

**Anas Shamsi, PhD**

E-mail: [anas.shamsi18@gmail.com](mailto:anas.shamsi18@gmail.com)

**Table S1**: List of deleterious mutations in Myosin-3 predicted through sequence-based tools.

| **S. No.** | **Mutation** | **SIFT** | **PolyPhen2** | **Mutation Assessor** | **FATHMM** |
| --- | --- | --- | --- | --- | --- |
|  | A151D | Deleterious | Possibly Damaging | Medium | Tolerated |
|  | A151G | Deleterious | Possibly Damaging | Low | Tolerated |
|  | A151T | Deleterious | Benign | Low | Tolerated |
|  | A162T | Deleterious | Benign | Medium | Damaging |
|  | A183S | Deleterious | Possibly Damaging | Low | Tolerated |
|  | A201V | Tolerated | Benign | Medium | Tolerated |
|  | A206T | Deleterious | Benign | Low | Tolerated |
|  | A206V | Deleterious | Benign | Neutral | Tolerated |
|  | A230G | Deleterious | Benign | High | Damaging |
|  | A234T | Deleterious | Possibly Damaging | High | Damaging |
|  | A260S | Deleterious | Benign | Neutral | Tolerated |
|  | A262G | Deleterious | Benign | Neutral | Tolerated |
|  | A262T | Deleterious | Probably Damaging | High | Damaging |
|  | A327G | Deleterious | Benign | Neutral | Tolerated |
|  | A336T | Deleterious | Probably Damaging | High | Damaging |
|  | A386T | Deleterious | Benign | Medium | Damaging |
|  | A424D | Deleterious | Benign | High | Damaging |
|  | A424S | Tolerated | Benign | Neutral | Tolerated |
|  | A519S | Deleterious | Possibly Damaging | Low | Tolerated |
|  | A519V | Deleterious | Benign | Low | Tolerated |
|  | A574V | Tolerated | Benign | Low | Tolerated |
|  | A576G | Deleterious | Benign | Medium | Tolerated |
|  | A584E | Deleterious | Probably Damaging | High | Damaging |
|  | A626G | Tolerated | Benign | Low | Tolerated |
|  | A628E | Deleterious | Benign | Low | Tolerated |
|  | A628T | Tolerated | Benign | Low | Tolerated |
|  | A628V | Deleterious | Benign | Medium | Tolerated |
|  | A630T | Deleterious | Benign | Low | Tolerated |
|  | A638T | Tolerated | Benign | Neutral | Tolerated |
|  | A684S | Deleterious | Benign | Neutral | Tolerated |
|  | A684V | Deleterious | Benign | Neutral | Tolerated |
|  | A728V | Deleterious | Possibly Damaging | Low | Tolerated |
|  | A730V | Deleterious | Benign | Neutral | Tolerated |
|  | A748V | Deleterious | Benign | Low | Tolerated |
|  | A768P | Deleterious | Probably Damaging | Medium | Damaging |
|  | A768V | Deleterious | Probably Damaging | Medium | Damaging |
|  | A92V | Deleterious | Benign | Low | Tolerated |
|  | C401R | Deleterious | Probably Damaging | Medium | Damaging |
|  | C521W | Deleterious | Probably Damaging | High | Damaging |
|  | C521Y | Deleterious | Probably Damaging | High | Damaging |
|  | D108Y | Deleterious | Possibly Damaging | Low | Tolerated |
|  | D169N | Deleterious | Benign | Neutral | Tolerated |
|  | D210N | Deleterious | Benign | Neutral | Tolerated |
|  | D219G | Deleterious | Probably Damaging | High | Damaging |
|  | D219H | Deleterious | Probably Damaging | Medium | Tolerated |
|  | D219Y | Deleterious | Probably Damaging | High | Damaging |
|  | D240Y | Deleterious | Possibly Damaging | High | Damaging |
|  | D263Y | Deleterious | Probably Damaging | Medium | Tolerated |
|  | D310H | Deleterious | Probably Damaging | Medium | Tolerated |
|  | D310N | Deleterious | Probably Damaging | Medium | Tolerated |
|  | D310Y | Deleterious | Probably Damaging | Medium | Damaging |
|  | D325G | Deleterious | Probably Damaging | Medium | Damaging |
|  | D338E | Deleterious | Benign | Low | Tolerated |
|  | D377G | Deleterious | Possibly Damaging | Medium | Damaging |
|  | D383G | Deleterious | Probably Damaging | Medium | Tolerated |
|  | D449V | Deleterious | Probably Damaging | Medium | Damaging |
|  | D462G | Deleterious | Probably Damaging | High | Damaging |
|  | D513E | Deleterious | Benign | Medium | Tolerated |
|  | D517A | Deleterious | Probably Damaging | High | Damaging |
|  | D517Y | Deleterious | Probably Damaging | High | Damaging |
|  | D546G | Deleterious | Probably Damaging | High | Damaging |
|  | D555E | Tolerated | Benign | Low | Tolerated |
|  | D629N | Tolerated | Benign | Low | Tolerated |
|  | D629Y | Deleterious | Benign | Medium | Damaging |
|  | D718N | Deleterious | Benign | Medium | Tolerated |
|  | D718Y | Deleterious | Possibly Damaging | High | Damaging |
|  | E138D | Deleterious | Possibly Damaging | Low | Tolerated |
|  | E138G | Deleterious | Benign | Medium | Damaging |
|  | E138K | Deleterious | Benign | Low | Tolerated |
|  | E150D | Deleterious | Probably Damaging | Low | Tolerated |
|  | E281K | Deleterious | Probably Damaging | High | Damaging |
|  | E297D | Tolerated | Benign | Low | Tolerated |
|  | E318D | Deleterious | Benign | Low | Tolerated |
|  | E318V | Deleterious | Benign | Neutral | Tolerated |
|  | E328D | Deleterious | Benign | Low | Tolerated |
|  | E375K | Deleterious | Probably Damaging | Medium | Tolerated |
|  | E375Q | Deleterious | Benign | Medium | Tolerated |
|  | E434Q | Deleterious | Possibly Damaging | Medium | Tolerated |
|  | E467A | Deleterious | Probably Damaging | High | Damaging |
|  | E500K | Deleterious | Probably Damaging | Medium | Damaging |
|  | E501K | Deleterious | Probably Damaging | High | Damaging |
|  | E508D | Tolerated | Benign | Neutral | Tolerated |
|  | E508G | Deleterious | Possibly Damaging | Medium | Damaging |
|  | E508K | Deleterious | Benign | Low | Tolerated |
|  | E523K | Deleterious | Possibly Damaging | Medium | Tolerated |
|  | E523Q | Deleterious | Possibly Damaging | Medium | Damaging |
|  | E526K | Deleterious | Possibly Damaging | High | Tolerated |
|  | E537D | Deleterious | Probably Damaging | Medium | Tolerated |
|  | E575K | Deleterious | Benign | Medium | Tolerated |
|  | E604G | Deleterious | Possibly Damaging | Low | Tolerated |
|  | E604K | Deleterious | Benign | Medium | Tolerated |
|  | E604Q | Deleterious | Possibly Damaging | Low | Tolerated |
|  | E654K | Deleterious | Possibly Damaging | Low | Tolerated |
|  | E678K | Deleterious | Probably Damaging | Medium | Tolerated |
|  | E686K | Deleterious | Benign | Medium | Tolerated |
|  | E701D | Deleterious | Benign | Medium | Damaging |
|  | E733D | Tolerated | Benign | Low | Tolerated |
|  | F156Y | Deleterious | Benign | Neutral | Tolerated |
|  | F165L | Deleterious | Benign | Low | Tolerated |
|  | F165Y | Deleterious | Benign | Neutral | Tolerated |
|  | F276C | Deleterious | Probably Damaging | Medium | Damaging |
|  | F287V | Deleterious | Probably Damaging | High | Damaging |
|  | F365C | Deleterious | Probably Damaging | High | Damaging |
|  | F365S | Deleterious | Probably Damaging | High | Damaging |
|  | F402I | Deleterious | Benign | Neutral | Tolerated |
|  | F437I | Deleterious | Probably Damaging | High | Damaging |
|  | F457L | Deleterious | Possibly Damaging | Medium | Tolerated |
|  | F457S | Deleterious | Probably Damaging | Low | Tolerated |
|  | F495L | Deleterious | Possibly Damaging | Medium | Tolerated |
|  | F532L | Deleterious | Benign | Neutral | Tolerated |
|  | F532S | Deleterious | Probably Damaging | High | Damaging |
|  | F532Y | Deleterious | Possibly Damaging | Medium | Damaging |
|  | F564V | Deleterious | Probably Damaging | Medium | Tolerated |
|  | F645C | Deleterious | Probably Damaging | High | Damaging |
|  | F652S | Deleterious | Probably Damaging | Medium | Damaging |
|  | G142S | Deleterious | Benign | Neutral | Tolerated |
|  | G145S | Deleterious | Probably Damaging | Medium | Damaging |
|  | G179R | Deleterious | Probably Damaging | High | Damaging |
|  | G182A | Deleterious | Possibly Damaging | High | Damaging |
|  | G182R | Deleterious | Probably Damaging | High | Damaging |
|  | G215R | Deleterious | Probably Damaging | Medium | Tolerated |
|  | G246D | Deleterious | Probably Damaging | High | Damaging |
|  | G341C | Deleterious | Probably Damaging | High | Damaging |
|  | G341R | Deleterious | Probably Damaging | High | Damaging |
|  | G355E | Deleterious | Probably Damaging | Medium | Tolerated |
|  | G361R | Deleterious | Probably Damaging | High | Damaging |
|  | G361W | Deleterious | Probably Damaging | High | Damaging |
|  | G378D | Deleterious | Probably Damaging | Low | Tolerated |
|  | G408R | Deleterious | Probably Damaging | Medium | Tolerated |
|  | G408V | Deleterious | Probably Damaging | Medium | Damaging |
|  | G465C | Deleterious | Probably Damaging | High | Damaging |
|  | G515R | Deleterious | Probably Damaging | Medium | Tolerated |
|  | G559A | Deleterious | Possibly Damaging | Medium | Tolerated |
|  | G572A | Deleterious | Benign | Low | Tolerated |
|  | G572D | Deleterious | Possibly Damaging | Medium | Tolerated |
|  | G608E | Deleterious | Benign | Neutral | Tolerated |
|  | G633R | Deleterious | Benign | Low | Tolerated |
|  | G683A | Deleterious | Probably Damaging | - | Tolerated |
|  | G683E | Deleterious | Probably Damaging | - | Tolerated |
|  | G717S | Deleterious | Benign | Neutral | Tolerated |
|  | G769D | Deleterious | Probably Damaging | Medium | Damaging |
|  | G769S | Deleterious | Probably Damaging | Medium | Damaging |
|  | H154Y | Deleterious | Probably Damaging | High | Damaging |
|  | H252L | Deleterious | Possibly Damaging | Medium | Tolerated |
|  | H285Y | Deleterious | Possibly Damaging | High | Damaging |
|  | H359Q | Deleterious | Probably Damaging | Medium | Damaging |
|  | H422Q | Deleterious | Benign | Neutral | Tolerated |
|  | H423R | Deleterious | Benign | Neutral | Tolerated |
|  | H492Y | Deleterious | Benign | Medium | Damaging |
|  | H557Q | Deleterious | Probably Damaging | Medium | Damaging |
|  | H620D | Tolerated | Benign | Neutral | Tolerated |
|  | H667D | Deleterious | Probably Damaging | Low | Tolerated |
|  | H667Q | Deleterious | Benign | Neutral | Tolerated |
|  | H754Y | Deleterious | Possibly Damaging | Medium | Tolerated |
|  | H96Q | Deleterious | Possibly Damaging | Low | Tolerated |
|  | H96R | Deleterious | Benign | Medium | Tolerated |
|  | H96Y | Deleterious | Benign | Neutral | Tolerated |
|  | I115F | Deleterious | Probably Damaging | High | Damaging |
|  | I115S | Deleterious | Probably Damaging | High | Damaging |
|  | I158S | Deleterious | Probably Damaging | Medium | Damaging |
|  | I158T | Deleterious | Probably Damaging | Medium | Damaging |
|  | I175F | Deleterious | Possibly Damaging | Medium | Damaging |
|  | I175L | Deleterious | Possibly Damaging | Medium | Damaging |
|  | I175V | Deleterious | Benign | Low | Tolerated |
|  | I199V | Deleterious | Benign | Neutral | Tolerated |
|  | I221T | Deleterious | Probably Damaging | High | Damaging |
|  | I222V | Deleterious | Benign | Neutral | Tolerated |
|  | I249F | Deleterious | Probably Damaging | Medium | Damaging |
|  | I264T | Deleterious | Probably Damaging | Medium | Damaging |
|  | I286V | Deleterious | Possibly Damaging | Low | Tolerated |
|  | I299V | Deleterious | Benign | Low | Tolerated |
|  | I304M | Deleterious | Probably Damaging | Medium | Tolerated |
|  | I304V | Deleterious | Benign | Neutral | Tolerated |
|  | I314M | Deleterious | Benign | Low | Tolerated |
|  | I314S | Deleterious | Possibly Damaging | Low | Tolerated |
|  | I324K | Deleterious | Probably Damaging | Medium | Tolerated |
|  | I324T | Deleterious | Probably Damaging | Medium | Tolerated |
|  | I337V | Tolerated | Benign | Low | Tolerated |
|  | I339M | Deleterious | Probably Damaging | Medium | Tolerated |
|  | I444V | Deleterious | Benign | Low | Tolerated |
|  | I458T | Deleterious | Probably Damaging | High | Damaging |
|  | I468L | Deleterious | Possibly Damaging | High | Damaging |
|  | I507T | Deleterious | Probably Damaging | High | Damaging |
|  | I507V | Deleterious | Possibly Damaging | Medium | Tolerated |
|  | I522M | Deleterious | Probably Damaging | High | Damaging |
|  | I525N | Deleterious | Probably Damaging | High | Damaging |
|  | I531V | Deleterious | Benign | Low | Tolerated |
|  | I674T | Deleterious | Probably Damaging | High | Damaging |
|  | I705V | Deleterious | Benign | Low | Tolerated |
|  | I731V | Deleterious | Benign | Neutral | Tolerated |
|  | I737T | Deleterious | Benign | Low | Tolerated |
|  | I752V | Tolerated | Benign | Low | Tolerated |
|  | K107N | Deleterious | Benign | Medium | Tolerated |
|  | K147N | Deleterious | Probably Damaging | Low | Tolerated |
|  | K147R | Deleterious | Benign | Neutral | Tolerated |
|  | K190T | Deleterious | Probably Damaging | High | Damaging |
|  | K208R | Deleterious | Benign | Low | Tolerated |
|  | K209E | Tolerated | Benign | Neutral | Tolerated |
|  | K209N | Deleterious | Benign | Low | Tolerated |
|  | K212R | Deleterious | Possibly Damaging | Low | Tolerated |
|  | K214N | Deleterious | Benign | Low | Tolerated |
|  | K258R | Deleterious | Probably Damaging | Low | Tolerated |
|  | K347E | Deleterious | Probably Damaging | Medium | Tolerated |
|  | K366M | Deleterious | Probably Damaging | Medium | Damaging |
|  | K366R | Deleterious | Probably Damaging | Medium | Tolerated |
|  | K398E | Deleterious | Probably Damaging | Medium | Tolerated |
|  | K435N | Deleterious | Probably Damaging | Low | Tolerated |
|  | K543M | Deleterious | Probably Damaging | Medium | Damaging |
|  | K568E | Deleterious | Possibly Damaging | Medium | Tolerated |
|  | K568R | Deleterious | Benign | Neutral | Tolerated |
|  | K571R | Deleterious | Possibly Damaging | Medium | Tolerated |
|  | K612N | Deleterious | Benign | Low | Tolerated |
|  | K636E | Deleterious | Possibly Damaging | Low | Tolerated |
|  | K636N | Deleterious | Possibly Damaging | Low | Tolerated |
|  | K636T | Deleterious | Possibly Damaging | Medium | Tolerated |
|  | K639E | Deleterious | Benign | Medium | Tolerated |
|  | K639Q | Deleterious | Probably Damaging | Low | Tolerated |
|  | K641R | Deleterious | Benign | Low | Tolerated |
|  | K720E | Deleterious | Possibly Damaging | Medium | Tolerated |
|  | K740R | Deleterious | Benign | Neutral | Tolerated |
|  | K745Q | Deleterious | Probably Damaging | Medium | Tolerated |
|  | K763N | Deleterious | Probably Damaging | High | Damaging |
|  | K767R | Deleterious | Benign | Neutral | Tolerated |
|  | L103P | Deleterious | Probably Damaging | High | Damaging |
|  | L106V | Deleterious | Probably Damaging | Medium | Damaging |
|  | L205P | Deleterious | Benign | Neutral | Tolerated |
|  | L227V | Deleterious | Benign | Neutral | Tolerated |
|  | L268P | Deleterious | Probably Damaging | High | Damaging |
|  | L291I | Deleterious | Benign | Medium | Damaging |
|  | L298F | Deleterious | Benign | Medium | Damaging |
|  | L298P | Deleterious | Probably Damaging | Medium | Damaging |
|  | L301V | Deleterious | Benign | Low | Tolerated |
|  | L302Q | Deleterious | Probably Damaging | High | Damaging |
|  | L331V | Deleterious | Benign | Low | Tolerated |
|  | L388M | Deleterious | Probably Damaging | Medium | Damaging |
|  | L396F | Deleterious | Benign | Low | Tolerated |
|  | L400F | Deleterious | Benign | Medium | Damaging |
|  | L428F | Deleterious | Probably Damaging | Medium | Damaging |
|  | L436F | Deleterious | Possibly Damaging | High | Damaging |
|  | L452F | Deleterious | Possibly Damaging | Medium | Tolerated |
|  | L518R | Deleterious | Probably Damaging | High | Tolerated |
|  | L558R | Deleterious | Benign | Medium | Tolerated |
|  | L617P | Deleterious | Possibly Damaging | High | Damaging |
|  | L621F | Deleterious | Possibly Damaging | Medium | Damaging |
|  | L621R | Deleterious | Probably Damaging | High | Damaging |
|  | L651H | Deleterious | Probably Damaging | Medium | Tolerated |
|  | L689F | Deleterious | Probably Damaging | Medium | Damaging |
|  | L689R | Deleterious | Probably Damaging | Medium | Tolerated |
|  | L691M | Deleterious | Possibly Damaging | Low | Damaging |
|  | L700P | Deleterious | Probably Damaging | High | Damaging |
|  | L715V | Deleterious | Benign | Low | Tolerated |
|  | L770F | Deleterious | Benign | Medium | Tolerated |
|  | L770S | Deleterious | Probably Damaging | Low | Tolerated |
|  | L771P | Deleterious | Probably Damaging | High | Damaging |
|  | M166V | Deleterious | Probably Damaging | High | Damaging |
|  | M213V | Deleterious | Benign | Neutral | Tolerated |
|  | M358V | Deleterious | Possibly Damaging | Medium | Tolerated |
|  | M363V | Deleterious | Benign | Low | Tolerated |
|  | M440T | Deleterious | Possibly Damaging | Low | Tolerated |
|  | M440V | Deleterious | Possibly Damaging | Neutral | Tolerated |
|  | M494T | Deleterious | Probably Damaging | Low | Tolerated |
|  | M494V | Deleterious | Possibly Damaging | Low | Tolerated |
|  | M529I | Deleterious | Benign | Low | Tolerated |
|  | M529V | Deleterious | Probably Damaging | Medium | Tolerated |
|  | M540I | Deleterious | Benign | Low | Tolerated |
|  | M540L | Deleterious | Benign | Low | Tolerated |
|  | M660I | Deleterious | Possibly Damaging | Medium | Tolerated |
|  | M660L | Deleterious | Benign | Medium | Tolerated |
|  | M685I | Deleterious | Benign | Low | Tolerated |
|  | M685V | Deleterious | Benign | Low | Tolerated |
|  | M777V | Deleterious | Benign | Low | Tolerated |
|  | M91I | Deleterious | Benign | Medium | Tolerated |
|  | M91L | Deleterious | Benign | Neutral | Tolerated |
|  | M91V | Deleterious | Possibly Damaging | Medium | Tolerated |
|  | M93V | Deleterious | Possibly Damaging | Medium | Tolerated |
|  | N105S | Deleterious | Probably Damaging | Medium | Damaging |
|  | N127D | Deleterious | Probably Damaging | High | Damaging |
|  | N127T | Deleterious | Probably Damaging | High | Damaging |
|  | N136H | Deleterious | Benign | Medium | Tolerated |
|  | N161S | Deleterious | Possibly Damaging | Low | Tolerated |
|  | N172D | Deleterious | Probably Damaging | Medium | Tolerated |
|  | N225S | Deleterious | Possibly Damaging | Medium | Damaging |
|  | N241D | Deleterious | Probably Damaging | High | Damaging |
|  | N241S | Deleterious | Possibly Damaging | High | Damaging |
|  | N483D | Deleterious | Probably Damaging | High | Damaging |
|  | N483S | Deleterious | Possibly Damaging | Medium | Damaging |
|  | N551D | Deleterious | Benign | Neutral | Tolerated |
|  | N551S | Deleterious | Benign | Neutral | Tolerated |
|  | N562K | Deleterious | Benign | Neutral | Tolerated |
|  | N562S | Tolerated | Benign | Neutral | Tolerated |
|  | N563I | Deleterious | Benign | Low | Tolerated |
|  | N563K | Deleterious | Benign | Neutral | Tolerated |
|  | N615S | Deleterious | Benign | Low | Tolerated |
|  | N657H | Deleterious | Probably Damaging | Medium | Tolerated |
|  | N677S | Deleterious | Probably Damaging | High | Damaging |
|  | N727D | Deleterious | Possibly Damaging | Low | Tolerated |
|  | N98S | Deleterious | Possibly Damaging | Low | Tolerated |
|  | P100S | Deleterious | Possibly Damaging | Low | Tolerated |
|  | P133A | Deleterious | Probably Damaging | Medium | Tolerated |
|  | P133L | Deleterious | Possibly Damaging | High | Damaging |
|  | P137T | Deleterious | Benign | Low | Tolerated |
|  | P153S | Deleterious | Probably Damaging | High | Damaging |
|  | P296A | Deleterious | Probably Damaging | Low | Tolerated |
|  | P296L | Deleterious | Probably Damaging | Medium | Tolerated |
|  | P296T | Deleterious | Possibly Damaging | Medium | Tolerated |
|  | P312L | Deleterious | Benign | Low | Tolerated |
|  | P344A | Tolerated | Benign | Low | Tolerated |
|  | P344R | Deleterious | Benign | Medium | Tolerated |
|  | P376Q | Deleterious | Possibly Damaging | Medium | Tolerated |
|  | P453S | Deleterious | Benign | Neutral | Tolerated |
|  | P528A | Deleterious | Probably Damaging | High | Damaging |
|  | P542R | Deleterious | Probably Damaging | High | Damaging |
|  | P542T | Deleterious | Probably Damaging | High | Damaging |
|  | P567L | Deleterious | Probably Damaging | High | Damaging |
|  | P601L | Deleterious | Probably Damaging | Low | Tolerated |
|  | P676A | Deleterious | Probably Damaging | High | Damaging |
|  | P682Q | Deleterious | Possibly Damaging | Medium | Damaging |
|  | Q289E | Deleterious | Probably Damaging | Medium | Damaging |
|  | Q289H | Deleterious | Probably Damaging | Medium | Damaging |
|  | Q316H | Deleterious | Benign | Medium | Tolerated |
|  | Q367R | Deleterious | Probably Damaging | Medium | Tolerated |
|  | Q420R | Deleterious | Probably Damaging | High | Damaging |
|  | Q446R | Deleterious | Benign | Neutral | Tolerated |
|  | Q556E | Deleterious | Benign | Low | Tolerated |
|  | Q556K | Deleterious | Benign | Low | Tolerated |
|  | Q721K | Deleterious | Possibly Damaging | Low | Tolerated |
|  | Q735P | Deleterious | Probably Damaging | Medium | Tolerated |
|  | Q756R | Deleterious | Possibly Damaging | Medium | Tolerated |
|  | R109G | Deleterious | Probably Damaging | High | Damaging |
|  | R109H | Deleterious | Probably Damaging | High | Damaging |
|  | R144G | Deleterious | Probably Damaging | Medium | Damaging |
|  | R144Q | Deleterious | Probably Damaging | Low | Tolerated |
|  | R148C | Deleterious | Probably Damaging | Medium | Damaging |
|  | R148G | Deleterious | Possibly Damaging | Medium | Damaging |
|  | R148H | Deleterious | Probably Damaging | Medium | Damaging |
|  | R148L | Deleterious | Probably Damaging | Low | Tolerated |
|  | R170C | Deleterious | Probably Damaging | Medium | Damaging |
|  | R170H | Deleterious | Possibly Damaging | Medium | Tolerated |
|  | R170L | Deleterious | Probably Damaging | Medium | Damaging |
|  | R170S | Deleterious | Probably Damaging | Medium | Tolerated |
|  | R191Q | Deleterious | Probably Damaging | Low | Tolerated |
|  | R191W | Deleterious | Probably Damaging | Medium | Damaging |
|  | R238G | Deleterious | Probably Damaging | High | Damaging |
|  | R244C | Deleterious | Probably Damaging | High | Damaging |
|  | R244G | Deleterious | Probably Damaging | High | Damaging |
|  | R244H | Deleterious | Probably Damaging | High | Damaging |
|  | R250Q | Deleterious | Probably Damaging | Low | Tolerated |
|  | R370Q | Deleterious | Probably Damaging | Medium | Tolerated |
|  | R404T | Deleterious | Probably Damaging | Low | Tolerated |
|  | R443C | Deleterious | Probably Damaging | High | Damaging |
|  | R443H | Deleterious | Probably Damaging | Medium | Damaging |
|  | R454S | Deleterious | Probably Damaging | Low | Tolerated |
|  | R653S | Deleterious | Probably Damaging | Medium | Tolerated |
|  | R672C | Deleterious | Probably Damaging | High | Damaging |
|  | R672H | Deleterious | Probably Damaging | High | Damaging |
|  | R695Q | Deleterious | Benign | Medium | Damaging |
|  | R695W | Deleterious | Probably Damaging | High | Damaging |
|  | R704C | Deleterious | Probably Damaging | High | Damaging |
|  | R704H | Deleterious | Probably Damaging | High | Damaging |
|  | R713S | Deleterious | Probably Damaging | High | Damaging |
|  | R778G | Deleterious | Probably Damaging | Medium | Damaging |
|  | R778Q | Deleterious | Possibly Damaging | High | Damaging |
|  | R778W | Deleterious | Probably Damaging | High | Damaging |
|  | S112T | Deleterious | Benign | Medium | Tolerated |
|  | S119L | Deleterious | Probably Damaging | Medium | Tolerated |
|  | S157F | Deleterious | Probably Damaging | Medium | Damaging |
|  | S174C | Deleterious | Probably Damaging | Medium | Tolerated |
|  | S174T | Deleterious | Probably Damaging | Medium | Damaging |
|  | S223N | Deleterious | Benign | Low | Tolerated |
|  | S223R | Deleterious | Benign | Neutral | Tolerated |
|  | S242F | Deleterious | Probably Damaging | High | Damaging |
|  | S261F | Deleterious | Probably Damaging | Medium | Damaging |
|  | S283N | Deleterious | Benign | Neutral | Tolerated |
|  | S283T | Deleterious | Benign | Low | Tolerated |
|  | S292C | Deleterious | Probably Damaging | Medium | Damaging |
|  | S315G | Deleterious | Probably Damaging | Medium | Tolerated |
|  | S315R | Deleterious | Probably Damaging | Medium | Tolerated |
|  | S335N | Deleterious | Benign | Neutral | Tolerated |
|  | S335R | Deleterious | Benign | Neutral | Tolerated |
|  | S335T | Tolerated | Benign | Neutral | Tolerated |
|  | S394L | Deleterious | Benign | Medium | Tolerated |
|  | S548T | Deleterious | Benign | Neutral | Tolerated |
|  | S579L | Deleterious | Possibly Damaging | Neutral | Tolerated |
|  | S590R | Deleterious | Benign | Low | Tolerated |
|  | S592L | Deleterious | Benign | Low | Tolerated |
|  | S613F | Deleterious | Possibly Damaging | High | Damaging |
|  | S632C | Deleterious | Possibly Damaging | Medium | Damaging |
|  | S632G | Tolerated | Benign | Neutral | Tolerated |
|  | S632N | Tolerated | Benign | Low | Tolerated |
|  | S643A | Deleterious | Benign | Neutral | Tolerated |
|  | S643F | Deleterious | Possibly Damaging | Medium | Damaging |
|  | S644F | Deleterious | Probably Damaging | Medium | Damaging |
|  | S729N | Deleterious | Benign | Neutral | Tolerated |
|  | S739N | Deleterious | Benign | Low | Tolerated |
|  | S749Y | Deleterious | Probably Damaging | Medium | Damaging |
|  | T117P | Deleterious | Probably Damaging | High | Damaging |
|  | T125A | Deleterious | Benign | Neutral | Tolerated |
|  | T178I | Deleterious | Probably Damaging | High | Tolerated |
|  | T178N | Deleterious | Probably Damaging | High | Damaging |
|  | T198I | Deleterious | Benign | Low | Damaging |
|  | T202S | Tolerated | Benign | Neutral | Tolerated |
|  | T236P | Deleterious | Probably Damaging | High | Damaging |
|  | T275S | Deleterious | Probably Damaging | Low | Tolerated |
|  | T305M | Deleterious | Probably Damaging | Medium | Tolerated |
|  | T306A | Deleterious | Benign | Low | Tolerated |
|  | T306P | Deleterious | Benign | Low | Tolerated |
|  | T333A | Deleterious | Benign | High | Damaging |
|  | T333I | Deleterious | Probably Damaging | Medium | Tolerated |
|  | T333R | Deleterious | Probably Damaging | High | Damaging |
|  | T343A | Deleterious | Benign | Medium | Tolerated |
|  | T354M | Deleterious | Possibly Damaging | Low | Tolerated |
|  | T379R | Deleterious | Probably Damaging | Medium | Tolerated |
|  | T385A | Tolerated | Benign | Neutral | Tolerated |
|  | T385I | Deleterious | Benign | Neutral | Tolerated |
|  | T385P | Deleterious | Benign | Low | Tolerated |
|  | T413S | Deleterious | Probably Damaging | Medium | Tolerated |
|  | T417I | Deleterious | Probably Damaging | High | Damaging |
|  | T417S | Deleterious | Possibly Damaging | Low | Tolerated |
|  | T442I | Deleterious | Benign | Low | Tolerated |
|  | T450M | Deleterious | Probably Damaging | Medium | Damaging |
|  | T482I | Deleterious | Probably Damaging | Medium | Tolerated |
|  | T510M | Deleterious | Possibly Damaging | Medium | Damaging |
|  | T545I | Deleterious | Possibly Damaging | High | Damaging |
|  | T545S | Deleterious | Benign | Neutral | Tolerated |
|  | T547N | Deleterious | Benign | Neutral | Tolerated |
|  | T547S | Deleterious | Benign | Neutral | Tolerated |
|  | T624M | Deleterious | Possibly Damaging | Low | Tolerated |
|  | T627K | Deleterious | Benign | Neutral | Tolerated |
|  | T627M | Deleterious | Possibly Damaging | Low | Damaging |
|  | T681A | Deleterious | Benign | Low | Tolerated |
|  | T755I | Deleterious | Possibly Damaging | Low | Tolerated |
|  | T762S | Deleterious | Probably Damaging | Medium | Tolerated |
|  | T773N | Deleterious | Benign | Low | Tolerated |
|  | T95M | Deleterious | Probably Damaging | Medium | Damaging |
|  | V102M | Deleterious | Probably Damaging | Medium | Damaging |
|  | V124A | Deleterious | Possibly Damaging | Medium | Damaging |
|  | V124I | Deleterious | Benign | Low | Tolerated |
|  | V126I | Deleterious | Benign | Neutral | Tolerated |
|  | V126L | Deleterious | Benign | Medium | Tolerated |
|  | V134G | Deleterious | Probably Damaging | Medium | Damaging |
|  | V134L | Deleterious | Benign | Neutral | Tolerated |
|  | V134M | Deleterious | Possibly Damaging | Medium | Tolerated |
|  | V139G | Deleterious | Probably Damaging | High | Damaging |
|  | V139L | Deleterious | Benign | Medium | Tolerated |
|  | V139M | Deleterious | Benign | Medium | Tolerated |
|  | V140M | Deleterious | Possibly Damaging | Low | Tolerated |
|  | V187K | Deleterious | Possibly Damaging | - | Tolerated |
|  | V237M | Deleterious | Possibly Damaging | Medium | Damaging |
|  | V357M | Deleterious | Probably Damaging | Medium | Damaging |
|  | V381A | Deleterious | Probably Damaging | - | Tolerated |
|  | V381L | Deleterious | Benign | Low | Tolerated |
|  | V412I | Deleterious | Probably Damaging | Low | Tolerated |
|  | V421A | Deleterious | Probably Damaging | Neutral | Tolerated |
|  | V421F | Deleterious | Probably Damaging | High | Damaging |
|  | V425L | Deleterious | Benign | Neutral | Tolerated |
|  | V496M | Deleterious | Probably Damaging | Medium | Damaging |
|  | V570G | Tolerated | Benign | Neutral | Tolerated |
|  | V570I | Tolerated | Benign | Neutral | Tolerated |
|  | V587L | Deleterious | Benign | High | Damaging |
|  | V587M | Deleterious | Probably Damaging | High | Damaging |
|  | V591F | Deleterious | Possibly Damaging | Medium | Damaging |
|  | V671M | Deleterious | Probably Damaging | High | Damaging |
|  | V690I | Deleterious | Benign | Low | Tolerated |
|  | V725M | Deleterious | Possibly Damaging | Low | Tolerated |
|  | W113L | Deleterious | Benign | Low | Tolerated |
|  | W113R | Deleterious | Probably Damaging | Low | Tolerated |
|  | W509G | Deleterious | Probably Damaging | High | Damaging |
|  | W594C | Deleterious | Probably Damaging | High | Damaging |
|  | Y116C | Deleterious | Probably Damaging | High | Damaging |
|  | Y116S | Deleterious | Probably Damaging | High | Damaging |
|  | Y129C | Deleterious | Possibly Damaging | High | Damaging |
|  | Y129H | Deleterious | Probably Damaging | High | Damaging |
|  | Y284D | Deleterious | Probably Damaging | High | Damaging |
|  | Y311C | Deleterious | Probably Damaging | High | Damaging |
|  | Y311S | Deleterious | Probably Damaging | High | Damaging |
|  | Y360C | Deleterious | Probably Damaging | Neutral | Tolerated |
|  | Y387C | Deleterious | Probably Damaging | Medium | Tolerated |
|  | Y471C | Deleterious | Possibly Damaging | Low | Tolerated |
|  | Y471F | Tolerated | Benign | Neutral | Tolerated |
|  | Y471H | Deleterious | Possibly Damaging | Neutral | Tolerated |
|  | Y502D | Deleterious | Probably Damaging | High | Damaging |
|  | Y554H | Deleterious | Possibly Damaging | Low | Tolerated |
|  | Y583S | Deleterious | Probably Damaging | High | Damaging |
|  | Y610H | Deleterious | Probably Damaging | Medium | Tolerated |
|  | Y622C | Deleterious | Possibly Damaging | Low | Tolerated |
|  | Y716C | Deleterious | Probably Damaging | High | Damaging |

**Table S2**: List of destabilizing mutations in Myosin-3 predicted through structure-based tools.

| **S. No.** | **Mutation** | **mCSM** | **PremPS** | **MAESTROweb** | **DynaMut2** |
| --- | --- | --- | --- | --- | --- |
|  | A151D | Destabilizing | 1.23 | -0.106 | -1.62 |
|  | A151G | Destabilizing | 1.21 | 0.035 | -1.45 |
|  | A151T | Destabilizing | 0.84 | 0.119 | -0.95 |
|  | A162T | Highly Destabilizing | 1.6 | 0.043 | -2.05 |
|  | A183S | Destabilizing | 1.07 | 0.378 | -0.75 |
|  | A201V | Destabilizing | -0.16 | -0.015 | -1.08 |
|  | A206T | Destabilizing | 0.13 | -0.138 | 0.15 |
|  | A206V | Destabilizing | -0.13 | -0.077 | -0.85 |
|  | A230G | Destabilizing | 1.52 | 0.147 | -2.07 |
|  | A234T | Highly Destabilizing | 1.82 | -0.216 | -1.68 |
|  | A260S | Destabilizing | 0.55 | -0.048 | -1.34 |
|  | A262G | Destabilizing | 1.1 | 0.12 | -2.12 |
|  | A262T | Destabilizing | 1.33 | -0.017 | -1.85 |
|  | A327G | Destabilizing | -0.09 | 0.064 | -1.36 |
|  | A336T | Destabilizing | 1.28 | 0.116 | -2.03 |
|  | A386T | Destabilizing | 1.2 | 0.125 | -1.95 |
|  | A424D | Highly Destabilizing | 1.2 | 0.099 | -1.62 |
|  | A424S | Destabilizing | 0.21 | 0.12 | -1.36 |
|  | A519S | Destabilizing | 0.67 | -0.304 | 0.29 |
|  | A519V | Destabilizing | 0.22 | -0.179 | -0.69 |
|  | A574V | Destabilizing | 0.18 | -0.063 | -1.24 |
|  | A576G | Destabilizing | 0.86 | 0.075 | -0.91 |
|  | A584E | Destabilizing | 0.99 | 0.029 | -0.72 |
|  | A626G | Destabilizing | 0.7 | -0.12 | -0.33 |
|  | A628E | Destabilizing | 0.64 | -0.393 | -0.73 |
|  | A628T | Destabilizing | 0.41 | -0.326 | 0.26 |
|  | A628V | Destabilizing | 0.14 | -0.366 | -1.08 |
|  | A630T | Destabilizing | 0.17 | -0.045 | 0.4 |
|  | A638T | Destabilizing | 0.2 | -0.169 | -0.06 |
|  | A684S | Destabilizing | 0.41 | 0 | -0.16 |
|  | A684V | Destabilizing | -0.7 | -0.037 | -0.83 |
|  | A728V | Destabilizing | 0.39 | -0.253 | -0.83 |
|  | A730V | Destabilizing | -0.05 | -0.061 | -0.88 |
|  | A748V | Destabilizing | 0.5 | -0.278 | -0.6 |
|  | A768P | Destabilizing | 1.04 | -0.086 | 0.14 |
|  | A768V | Destabilizing | 0.56 | -0.09 | -0.74 |
|  | A92V | Destabilizing | 0.54 | -0.02 | -0.65 |
|  | C401R | Destabilizing | 1.71 | 0.083 | -1.18 |
|  | C521W | Destabilizing | 1.35 | -0.154 | -1.25 |
|  | C521Y | Destabilizing | 0.92 | -0.107 | -0.83 |
|  | D108Y | Destabilizing | 0.23 | -0.199 | -0.44 |
|  | D169N | Destabilizing | -0.19 | -0.174 | -0.24 |
|  | D210N | Stabilizing | -0.31 | -0.047 | -0.15 |
|  | D219G | Destabilizing | 0.61 | 0.072 | -0.3 |
|  | D219H | Destabilizing | 0.62 | -0.153 | -1.11 |
|  | D219Y | Destabilizing | 0.32 | -0.363 | -0.71 |
|  | D240Y | Destabilizing | 0 | -0.424 | -0.35 |
|  | D263Y | Destabilizing | -0.08 | -0.253 | -0.56 |
|  | D310H | Destabilizing | 1.02 | -0.087 | 0.32 |
|  | D310N | Destabilizing | 0.67 | -0.156 | -0.08 |
|  | D310Y | Stabilizing | 0.39 | -0.248 | 0.44 |
|  | D325G | Destabilizing | 0.52 | 0.281 | 0.19 |
|  | D338E | Destabilizing | 0.53 | -0.279 | -0.67 |
|  | D377G | Destabilizing | 0.6 | 0.203 | 0.19 |
|  | D383G | Destabilizing | 0.66 | 0.085 | 0.23 |
|  | D449V | Stabilizing | 0.5 | 0.048 | -0.01 |
|  | D462G | Destabilizing | 0.78 | 0.131 | -0.78 |
|  | D513E | Destabilizing | 0.31 | -0.081 | 0.01 |
|  | D517A | Destabilizing | 0.31 | 0.147 | 0.33 |
|  | D517Y | Stabilizing | 0.06 | -0.175 | 0.38 |
|  | D546G | Destabilizing | 0.24 | 0.189 | -0.26 |
|  | D555E | Destabilizing | 0.24 | -0.315 | -0.03 |
|  | D629N | Stabilizing | 0.19 | 0.011 | 0.75 |
|  | D629Y | Stabilizing | 0.17 | -0.087 | 0.4 |
|  | D718N | Destabilizing | 0.48 | -0.199 | -0.68 |
|  | D718Y | Stabilizing | 0.34 | -0.256 | 0.22 |
|  | E138D | Destabilizing | 0.24 | -0.2 | -0.19 |
|  | E138G | Destabilizing | 0.44 | -0.009 | 0.1 |
|  | E138K | Stabilizing | -0.23 | -0.144 | 0.19 |
|  | E150D | Destabilizing | 0.38 | -0.199 | 0.13 |
|  | E281K | Destabilizing | 1.2 | 0.12 | -1.06 |
|  | E297D | Destabilizing | 0.41 | 0.089 | -0.21 |
|  | E318D | Destabilizing | 0.68 | 0.011 | -0.76 |
|  | E318V | Destabilizing | -0.01 | -0.026 | -0.38 |
|  | E328D | Destabilizing | 0.31 | -0.212 | 0.03 |
|  | E375K | Destabilizing | 0.76 | 0.033 | 0.04 |
|  | E375Q | Destabilizing | 0.59 | -0.148 | -0.34 |
|  | E434Q | Destabilizing | 0.87 | -0.076 | -0.24 |
|  | E467A | Destabilizing | 1.54 | 0.112 | -0.3 |
|  | E500K | Destabilizing | 0.7 | -0.19 | -0.47 |
|  | E501K | Destabilizing | 1 | 0.083 | -0.43 |
|  | E508D | Destabilizing | 0.16 | 0.044 | -0.13 |
|  | E508G | Destabilizing | 0.52 | 0.21 | 0.18 |
|  | E508K | Stabilizing | 0.09 | -0.062 | 0.33 |
|  | E523K | Destabilizing | 0.8 | -0.149 | -0.01 |
|  | E523Q | Destabilizing | 0.73 | -0.129 | -0.09 |
|  | E526K | Stabilizing | 0.88 | -0.039 | 0.07 |
|  | E537D | Destabilizing | 0.62 | -0.089 | -0.44 |
|  | E575K | Stabilizing | 0.05 | -0.042 | 0.71 |
|  | E604G | Destabilizing | 0.71 | 0.053 | -0.07 |
|  | E604K | Stabilizing | 0.54 | -0.101 | 0.29 |
|  | E604Q | Destabilizing | 0.12 | -0.217 | -0.06 |
|  | E654K | Destabilizing | -0.05 | -0.083 | -0.15 |
|  | E678K | Stabilizing | 0.45 | 0.087 | 0.26 |
|  | E686K | Destabilizing | 0.73 | 0.012 | 0.04 |
|  | E701D | Destabilizing | 1.02 | 0.128 | -0.72 |
|  | E733D | Destabilizing | 0.14 | -0.001 | -0.27 |
|  | F156Y | Destabilizing | 1 | 0.036 | -0.04 |
|  | F165L | Destabilizing | 1.12 | -0.075 | -1.06 |
|  | F165Y | Destabilizing | 0.05 | -0.22 | 0.05 |
|  | F276C | Destabilizing | 1.06 | 0.033 | 0.72 |
|  | F287V | Destabilizing | 1.88 | 0.276 | -2.33 |
|  | F365C | Destabilizing | 2.12 | 0.006 | -1.7 |
|  | F365S | Highly Destabilizing | 2.34 | 0.245 | -3.21 |
|  | F402I | Destabilizing | 1.29 | -0.102 | -1.22 |
|  | F437I | Destabilizing | 1.84 | 0.058 | 0.64 |
|  | F457L | Destabilizing | 1.37 | -0.092 | -2.01 |
|  | F457S | Highly Destabilizing | 1.85 | 0.089 | -2.9 |
|  | F495L | Destabilizing | 1.39 | 0.137 | -2.16 |
|  | F532L | Destabilizing | 0.86 | -0.001 | 0.36 |
|  | F532S | Highly Destabilizing | 2.46 | 0.257 | -3.23 |
|  | F532Y | Destabilizing | 1.23 | -0.053 | -0.18 |
|  | F564V | Destabilizing | 1.54 | 0.225 | -1.45 |
|  | F645C | Destabilizing | 1.03 | -0.15 | 1 |
|  | F652S | Highly Destabilizing | 2.22 | 0.283 | -3.46 |
|  | G142S | Destabilizing | -0.2 | -0.215 | -0.62 |
|  | G145S | Destabilizing | 0.9 | -0.008 | -0.4 |
|  | G179R | Destabilizing | 0.99 | -0.129 | -0.81 |
|  | G182A | Destabilizing | 1.08 | 0.082 | -0.37 |
|  | G182R | Destabilizing | 1.17 | -0.079 | -0.57 |
|  | G215R | Destabilizing | 1.25 | -0.118 | -0.04 |
|  | G246D | Destabilizing | 1.36 | 0.019 | -0.77 |
|  | G341C | Destabilizing | 0.67 | -0.151 | -0.71 |
|  | G341R | Destabilizing | 0.97 | -0.107 | -0.95 |
|  | G355E | Highly Destabilizing | 1.51 | -0.066 | -1.61 |
|  | G361R | Destabilizing | 0.98 | -0.235 | -1.3 |
|  | G361W | Destabilizing | 0.67 | -0.381 | -1.29 |
|  | G378D | Destabilizing | 0.68 | -0.158 | -0.07 |
|  | G408R | Destabilizing | 1.13 | -0.188 | -0.36 |
|  | G408V | Destabilizing | 1.14 | -0.172 | -1.02 |
|  | G465C | Destabilizing | 1.36 | -0.031 | -0.71 |
|  | G515R | Destabilizing | 1.09 | -0.229 | -0.08 |
|  | G559A | Destabilizing | 0.97 | -0.273 | -0.22 |
|  | G572A | Destabilizing | 0.72 | -0.109 | -0.42 |
|  | G572D | Destabilizing | 0.73 | -0.11 | -0.18 |
|  | G608E | Destabilizing | 0.87 | -0.177 | 0.17 |
|  | G633R | Destabilizing | 0.94 | -0.17 | -0.7 |
|  | G683A | Destabilizing | 0.92 | -0.034 | -0.06 |
|  | G683E | Destabilizing | 1.13 | -0.112 | -0.34 |
|  | G717S | Destabilizing | 0.46 | -0.177 | -0.75 |
|  | G769D | Destabilizing | 1.15 | -0.064 | -0.81 |
|  | G769S | Destabilizing | 0.91 | 0.047 | -0.42 |
|  | H154Y | Destabilizing | 0.42 | 0.067 | -0.48 |
|  | H252L | Destabilizing | 0.38 | -0.223 | -0.31 |
|  | H285Y | Destabilizing | 0.75 | 0.006 | -0.02 |
|  | H359Q | Destabilizing | 1.47 | 0.034 | -1.21 |
|  | H422Q | Destabilizing | 0.64 | -0.229 | 0.14 |
|  | H423R | Destabilizing | 1 | -0.315 | 0.4 |
|  | H492Y | Stabilizing | 0.36 | -0.291 | 1.39 |
|  | H557Q | Destabilizing | 1.43 | 0.21 | -1.08 |
|  | H620D | Destabilizing | 0.12 | -0.088 | 0.15 |
|  | H667D | Destabilizing | 1.06 | -0.072 | 0.25 |
|  | H667Q | Stabilizing | 0.75 | -0.145 | 0.56 |
|  | H754Y | Stabilizing | 0.26 | -0.385 | 1.21 |
|  | H96Q | Stabilizing | 0.72 | -0.006 | 0.43 |
|  | H96R | Stabilizing | 0.77 | 0.128 | 0.23 |
|  | H96Y | Stabilizing | -0.38 | -0.045 | 1.46 |
|  | I115F | Destabilizing | 0.65 | 0.117 | -1.51 |
|  | I115S | Highly Destabilizing | 1.89 | 0.526 | -3.07 |
|  | I158S | Highly Destabilizing | 1.95 | 0.318 | -2.62 |
|  | I158T | Highly Destabilizing | 1.81 | 0.223 | -1.81 |
|  | I175F | Destabilizing | 0.67 | 0.005 | -2.42 |
|  | I175L | Destabilizing | 1.07 | -0.109 | -0.77 |
|  | I175V | Destabilizing | 0.76 | 0.075 | -1.09 |
|  | I199V | Destabilizing | 0.5 | 0.09 | -1.12 |
|  | I221T | Highly Destabilizing | 1.91 | 0.374 | -2.92 |
|  | I222V | Destabilizing | 0.41 | -0.089 | -1.09 |
|  | I249F | Destabilizing | 1 | -0.076 | -1.51 |
|  | I264T | Highly Destabilizing | 1.9 | 0.181 | -3.27 |
|  | I286V | Destabilizing | 1.13 | 0.153 | -1.16 |
|  | I299V | Destabilizing | 0.81 | 0.072 | -1.2 |
|  | I304M | Destabilizing | 1.08 | 0.193 | -0.11 |
|  | I304V | Destabilizing | 0.59 | 0.279 | -0.95 |
|  | I314M | Destabilizing | 1.25 | 0.041 | -1.09 |
|  | I314S | Highly Destabilizing | 2.35 | 0.376 | -3.69 |
|  | I324K | Destabilizing | 1.68 | -0.041 | 0.51 |
|  | I324T | Destabilizing | 1.43 | 0.158 | 0.21 |
|  | I337V | Destabilizing | 1.1 | 0.142 | -1.08 |
|  | I339M | Destabilizing | 0.78 | -0.168 | 0.02 |
|  | I444V | Destabilizing | 1.35 | 0.12 | -1.1 |
|  | I458T | Highly Destabilizing | 2.57 | 0.166 | -3.28 |
|  | I468L | Destabilizing | 0.26 | -0.056 | -0.62 |
|  | I507T | Highly Destabilizing | 1.73 | 0.323 | -1.24 |
|  | I507V | Destabilizing | 0.96 | 0.237 | -0.9 |
|  | I522M | Destabilizing | 1.15 | 0.019 | -0.95 |
|  | I525N | Highly Destabilizing | 2.85 | 0.325 | -2.73 |
|  | I531V | Destabilizing | 1.34 | 0.249 | 0.19 |
|  | I674T | Highly Destabilizing | 2.08 | 0.265 | -3.38 |
|  | I705V | Destabilizing | 1.09 | 0.159 | -1.2 |
|  | I731V | Destabilizing | 0.86 | 0.084 | -0.71 |
|  | I737T | Destabilizing | 1.5 | 0.224 | -1.35 |
|  | I752V | Destabilizing | 0.77 | 0.118 | -1.19 |
|  | K107N | Destabilizing | 1.09 | -0.115 | -0.96 |
|  | K147N | Stabilizing | 0.75 | 0.054 | 0.23 |
|  | K147R | Destabilizing | 0.26 | -0.094 | -0.43 |
|  | K190T | Destabilizing | 0.73 | -0.169 | -0.69 |
|  | K208R | Stabilizing | -0.02 | -0.158 | 0.09 |
|  | K209E | Destabilizing | -0.07 | -0.127 | -0.25 |
|  | K209N | Stabilizing | 0.11 | -0.067 | 0.18 |
|  | K212R | Destabilizing | 0.13 | -0.047 | -0.24 |
|  | K214N | Stabilizing | 0.28 | -0.013 | 0.26 |
|  | K258R | Destabilizing | 0.53 | -0.101 | -0.33 |
|  | K347E | Destabilizing | 1.34 | -0.105 | -0.44 |
|  | K366M | Stabilizing | 0.47 | -0.251 | 0.37 |
|  | K366R | Destabilizing | 0.49 | -0.273 | 0.16 |
|  | K398E | Stabilizing | 0.51 | -0.255 | -0.59 |
|  | K435N | Destabilizing | 1.24 | -0.141 | -0.77 |
|  | K543M | Destabilizing | 0.09 | 0.029 | -0.12 |
|  | K568E | Destabilizing | 0.36 | -0.007 | -0.39 |
|  | K568R | Destabilizing | -0.26 | -0.149 | -0.13 |
|  | K571R | Destabilizing | 0.01 | -0.091 | -0.26 |
|  | K612N | Stabilizing | 0.26 | -0.244 | 0.42 |
|  | K636E | Destabilizing | 0.19 | -0.029 | -0.4 |
|  | K636N | Stabilizing | 0.19 | -0.061 | 0.1 |
|  | K636T | Destabilizing | 0.04 | -0.039 | -0.31 |
|  | K639E | Destabilizing | 0.34 | -0.078 | -0.48 |
|  | K639Q | Destabilizing | 0.17 | -0.058 | -0.23 |
|  | K641R | Destabilizing | 0.04 | -0.149 | -0.3 |
|  | K720E | Destabilizing | 1.26 | -0.281 | -0.44 |
|  | K740R | Destabilizing | -0.01 | -0.289 | -0.33 |
|  | K745Q | Destabilizing | 0.6 | -0.272 | -0.29 |
|  | K763N | Destabilizing | 1.55 | -0.075 | -1.58 |
|  | K767R | Destabilizing | 0.34 | -0.064 | -0.05 |
|  | L103P | Destabilizing | 2.12 | 0.466 | -1.86 |
|  | L106V | Destabilizing | 1.75 | 0.263 | -2.08 |
|  | L205P | Destabilizing | -0.06 | -0.091 | -0.02 |
|  | L227V | Destabilizing | 0.95 | 0.16 | -2.26 |
|  | L268P | Destabilizing | 1.91 | 0.327 | -0.21 |
|  | L291I | Destabilizing | 0.98 | 0.087 | -0.57 |
|  | L298F | Destabilizing | 1.09 | 0.129 | -1.28 |
|  | L298P | Destabilizing | 2.04 | 0.312 | -1.34 |
|  | L301V | Destabilizing | 0.82 | -0.109 | -0.5 |
|  | L302Q | Highly Destabilizing | 2.58 | 0.293 | -3.31 |
|  | L331V | Destabilizing | 0.68 | -0.077 | -0.7 |
|  | L388M | Destabilizing | 1.02 | 0.081 | -1.06 |
|  | L396F | Destabilizing | 0.42 | 0.149 | -1.76 |
|  | L400F | Destabilizing | 1 | 0.162 | -1.84 |
|  | L428F | Destabilizing | 1.36 | 0.155 | -1.87 |
|  | L436F | Destabilizing | 0.66 | 0.158 | -1.01 |
|  | L452F | Destabilizing | 0.37 | -0.092 | -0.39 |
|  | L518R | Destabilizing | 1.57 | 0.157 | -0.28 |
|  | L558R | Destabilizing | 1.36 | -0.108 | 0.5 |
|  | L617P | Destabilizing | 1.69 | 0.258 | -1.38 |
|  | L621F | Destabilizing | 0.62 | 0.122 | -1.57 |
|  | L621R | Highly Destabilizing | 2 | 0.107 | -1.88 |
|  | L651H | Destabilizing | 1.39 | -0.197 | -0.39 |
|  | L689F | Destabilizing | 0.77 | -0.023 | -1.75 |
|  | L689R | Destabilizing | 1.81 | -0.136 | -0.21 |
|  | L691M | Destabilizing | 0.32 | 0.033 | 0.35 |
|  | L700P | Destabilizing | 1.72 | 0.219 | -1.18 |
|  | L715V | Destabilizing | 0.61 | -0.145 | -0.26 |
|  | L770F | Destabilizing | 0.65 | 0.073 | -0.73 |
|  | L770S | Highly Destabilizing | 1.84 | 0.264 | -3.06 |
|  | L771P | Destabilizing | 1.86 | 0.142 | -0.94 |
|  | M166V | Destabilizing | 1.68 | 0.188 | -1.18 |
|  | M213V | Stabilizing | 0.46 | 0.051 | 0.4 |
|  | M358V | Destabilizing | 1.76 | 0.149 | -1.7 |
|  | M363V | Highly Destabilizing | 1.03 | 0.242 | -0.51 |
|  | M440T | Destabilizing | 2.05 | 0.418 | 0.49 |
|  | M440V | Stabilizing | 1.51 | 0.218 | 0.56 |
|  | M494T | Destabilizing | 1.65 | 0.26 | -1.85 |
|  | M494V | Destabilizing | 1.43 | 0.175 | -1.68 |
|  | M529I | Destabilizing | 0.32 | -0.017 | -0.34 |
|  | M529V | Destabilizing | 0.88 | 0.039 | -0.31 |
|  | M540I | Destabilizing | 0.36 | -0.175 | -0.12 |
|  | M540L | Destabilizing | 0.48 | -0.161 | -0.21 |
|  | M660I | Destabilizing | 1.11 | 0.065 | -0.77 |
|  | M660L | Destabilizing | 1.28 | -0.027 | -0.65 |
|  | M685I | Highly Destabilizing | 0.79 | 0.056 | -1.19 |
|  | M685V | Highly Destabilizing | 1.22 | 0.065 | -0.41 |
|  | M777V | Destabilizing | 0.71 | -0.199 | -0.32 |
|  | M91I | Destabilizing | 1.25 | 0.199 | -0.36 |
|  | M91L | Destabilizing | 1.06 | 0.177 | -0.38 |
|  | M91V | Destabilizing | 1.71 | 0.19 | -1.06 |
|  | M93V | Destabilizing | 0.5 | 0.078 | -0.23 |
|  | N105S | Destabilizing | 1.26 | 0.133 | prediction |
|  | N127D | Destabilizing | 1.23 | -0.084 | -0.64 |
|  | N127T | Destabilizing | 0.93 | -0.253 | -0.7 |
|  | N136H | Destabilizing | 0.7 | -0.041 | 0.06 |
|  | N161S | Destabilizing | 0.77 | 0.076 | -0.03 |
|  | N172D | Destabilizing | 0.97 | -0.018 | -0.32 |
|  | N225S | Destabilizing | 1.13 | 0.184 | -1.09 |
|  | N241D | Destabilizing | 1.05 | -0.142 | -0.24 |
|  | N241S | Destabilizing | 0.83 | -0.071 | -0.84 |
|  | N483D | Highly Destabilizing | 1.39 | -0.007 | 0.4 |
|  | N483S | Destabilizing | 1.31 | 0.148 | -1.05 |
|  | N551D | Destabilizing | 0.51 | -0.207 | -1.01 |
|  | N551S | Destabilizing | -0.12 | -0.053 | -0.08 |
|  | N562K | Stabilizing | -0.02 | -0.109 | -0.09 |
|  | N562S | Stabilizing | -0.27 | 0.016 | 0.21 |
|  | N563I | Stabilizing | 0.42 | -0.114 | 0.35 |
|  | N563K | Destabilizing | 0.5 | -0.064 | 0.27 |
|  | N615S | Destabilizing | 0.45 | 0.237 | -0.07 |
|  | N657H | Destabilizing | 0.54 | -0.152 | -0.24 |
|  | N677S | Destabilizing | 1.22 | 0.136 | -1.34 |
|  | N727D | Stabilizing | 0.93 | 0.004 | -0.86 |
|  | N98S | Destabilizing | 0.24 | -0.026 | 0.08 |
|  | P100S | Destabilizing | 1.08 | -0.229 | 0.44 |
|  | P133A | Destabilizing | 0.92 | 0.022 | -0.91 |
|  | P133L | Destabilizing | 0.45 | -0.091 | -0.44 |
|  | P137T | Destabilizing | 0.46 | -0.151 | -0.47 |
|  | P153S | Highly Destabilizing | 1.17 | 0.246 | -0.17 |
|  | P296A | Destabilizing | 1.1 | -0.116 | -1.93 |
|  | P296L | Destabilizing | 0.61 | -0.218 | -0.18 |
|  | P296T | Destabilizing | 1.08 | -0.245 | -0.51 |
|  | P312L | Destabilizing | 0.41 | -0.383 | -0.2 |
|  | P344A | Destabilizing | 0.17 | -0.16 | -0.47 |
|  | P344R | Stabilizing | 0.44 | -0.296 | -0.35 |
|  | P376Q | Destabilizing | 1.05 | -0.197 | 0.33 |
|  | P453S | Destabilizing | 0.93 | 0.032 | -0.36 |
|  | P528A | Destabilizing | 0.73 | 0.028 | -0.1 |
|  | P542R | Destabilizing | 0.83 | -0.181 | -0.44 |
|  | P542T | Destabilizing | 0.71 | -0.096 | 0.22 |
|  | P567L | Destabilizing | 0.59 | -0.03 | -0.26 |
|  | P601L | Destabilizing | 0.6 | -0.202 | -0.45 |
|  | P676A | Highly Destabilizing | 1.49 | 0.14 | -0.36 |
|  | P682Q | Destabilizing | 1.16 | -0.112 | -2.5 |
|  | Q289E | Highly Destabilizing | 1.5 | 0.126 | 0.12 |
|  | Q289H | Destabilizing | 1.35 | 0.125 | -0.28 |
|  | Q316H | Destabilizing | 0.42 | -0.045 | -0.85 |
|  | Q367R | Destabilizing | 0.38 | -0.148 | -0.92 |
|  | Q420R | Destabilizing | 0.9 | 0.009 | -0.2 |
|  | Q446R | Stabilizing | -0.02 | -0.247 | -0.12 |
|  | Q556E | Destabilizing | 0.77 | -0.14 | 0.15 |
|  | Q556K | Destabilizing | 0.62 | -0.112 | 0.19 |
|  | Q721K | Stabilizing | 0.14 | -0.189 | 0.12 |
|  | Q735P | Stabilizing | 0.09 | 0.148 | -0.03 |
|  | Q756R | Destabilizing | 0.89 | -0.242 | 0.02 |
|  | R109G | Destabilizing | 1.96 | 0.157 | -0.2 |
|  | R109H | Destabilizing | 1.96 | 0.083 | -1.28 |
|  | R144G | Destabilizing | 1.04 | 0.183 | -1.47 |
|  | R144Q | Destabilizing | 0.45 | 0.053 | -0.42 |
|  | R148C | Destabilizing | 0.89 | -0.1 | -0.41 |
|  | R148G | Destabilizing | 1.61 | 0.061 | 0.68 |
|  | R148H | Destabilizing | 1.7 | -0.163 | -0.45 |
|  | R148L | Stabilizing | 0.78 | -0.151 | -1.51 |
|  | R170C | Stabilizing | 0.23 | -0.033 | 1.38 |
|  | R170H | Destabilizing | 0.35 | 0.028 | 0.8 |
|  | R170L | Stabilizing | 0.32 | 0.027 | -0.55 |
|  | R170S | Stabilizing | 0.55 | -0.133 | 1.17 |
|  | R191Q | Destabilizing | 1.34 | -0.065 | 0.03 |
|  | R191W | Destabilizing | 1.03 | -0.113 | -0.79 |
|  | R238G | Destabilizing | 1.39 | 0.292 | -0.61 |
|  | R244C | Highly Destabilizing | 0.79 | 0.151 | -1.38 |
|  | R244G | Highly Destabilizing | 1.7 | 0.441 | -2.02 |
|  | R244H | Highly Destabilizing | 1.82 | 0.188 | -1.98 |
|  | R250Q | Destabilizing | 1.26 | -0.038 | -1.11 |
|  | R370Q | Stabilizing | 0.54 | -0.479 | -0.75 |
|  | R404T | Destabilizing | 0.54 | -0.157 | -0.36 |
|  | R443C | Destabilizing | 0.73 | 0.074 | -0.38 |
|  | R443H | Highly Destabilizing | 1.23 | -0.082 | -1.53 |
|  | R454S | Destabilizing | 1.54 | 0.142 | -1.4 |
|  | R653S | Highly Destabilizing | 1.64 | 0.179 | -1 |
|  | R672C | Highly Destabilizing | 0.8 | -0.078 | -1.9 |
|  | R672H | Highly Destabilizing | 1.89 | -0.066 | -2.43 |
|  | R695Q | Destabilizing | 0.78 | -0.242 | -1.28 |
|  | R695W | Destabilizing | 0.38 | -0.253 | -0.51 |
|  | R704C | Destabilizing | 0.88 | -0.135 | 0.12 |
|  | R704H | Destabilizing | 1.59 | -0.147 | -1.31 |
|  | R713S | Destabilizing | 1.35 | -0.044 | -1.37 |
|  | R778G | Destabilizing | 1.56 | -0.096 | -0.68 |
|  | R778Q | Destabilizing | 1.51 | -0.191 | -1.61 |
|  | R778W | Destabilizing | 0.63 | -0.295 | -0.76 |
|  | S112T | Destabilizing | 0.25 | -0.113 | -0.51 |
|  | S119L | Destabilizing | 0.95 | -0.486 | -0.41 |
|  | S157F | Destabilizing | 0.89 | -0.212 | 0.02 |
|  | S174C | Destabilizing | 1.22 | -0.338 | -1.09 |
|  | S174T | Destabilizing | 0.78 | -0.335 | -0.46 |
|  | S223N | Destabilizing | 0.76 | -0.009 | -0.4 |
|  | S223R | Destabilizing | 0.36 | -0.314 | -0.67 |
|  | S242F | Destabilizing | 0.41 | -0.171 | -0.71 |
|  | S261F | Destabilizing | 0.89 | -0.442 | -0.32 |
|  | S283N | Destabilizing | 0.35 | 0.231 | -0.15 |
|  | S283T | Destabilizing | 0.3 | 0.156 | -0.86 |
|  | S292C | Destabilizing | 0.68 | -0.202 | -0.54 |
|  | S315G | Destabilizing | 0.98 | 0.017 | -0.51 |
|  | S315R | Destabilizing | 0.71 | -0.124 | -0.45 |
|  | S335N | Destabilizing | 0.3 | -0.253 | -0.16 |
|  | S335R | Destabilizing | 0.66 | -0.379 | 0.07 |
|  | S335T | Destabilizing | 0.12 | -0.227 | -0.08 |
|  | S394L | Destabilizing | 0.54 | -0.275 | -0.28 |
|  | S548T | Destabilizing | -0.45 | -0.151 | -0.53 |
|  | S579L | Stabilizing | 0.7 | -0.268 | -0.23 |
|  | S590R | Destabilizing | 0.71 | -0.05 | 0.15 |
|  | S592L | Destabilizing | -0.04 | -0.088 | -0.08 |
|  | S613F | Destabilizing | 0.47 | -0.08 | -0.54 |
|  | S632C | Destabilizing | 0.31 | -0.077 | -1.02 |
|  | S632G | Destabilizing | 0.02 | 0.035 | -0.24 |
|  | S632N | Destabilizing | 0.15 | -0.127 | -0.49 |
|  | S643A | Destabilizing | 0.05 | -0.168 | -0.48 |
|  | S643F | Destabilizing | 0.62 | -0.277 | -0.17 |
|  | S644F | Destabilizing | 0.63 | -0.254 | -0.8 |
|  | S729N | Destabilizing | 0.23 | -0.3 | -1.11 |
|  | S739N | Destabilizing | 0.57 | -0.051 | 0.06 |
|  | S749Y | Destabilizing | 0.51 | -0.318 | -0.5 |
|  | T117P | Destabilizing | 1.49 | -0.09 | -0.59 |
|  | T125A | Destabilizing | 0.96 | 0.065 | -1.35 |
|  | T178I | Destabilizing | 0.97 | -0.418 | -1.02 |
|  | T178N | Destabilizing | 1.19 | -0.011 | -0.39 |
|  | T198I | Destabilizing | -0.06 | -0.306 | -0.77 |
|  | T202S | Destabilizing | 0.19 | 0.003 | -0.31 |
|  | T236P | Stabilizing | 1.52 | 0.111 | -0.35 |
|  | T275S | Destabilizing | 0.91 | 0.085 | 0.35 |
|  | T305M | Destabilizing | 0.93 | -0.062 | -0.61 |
|  | T306A | Destabilizing | 0.76 | 0.208 | 0.29 |
|  | T306P | Destabilizing | 0.8 | 0.032 | -0.09 |
|  | T333A | Destabilizing | 1.46 | 0.004 | 0.03 |
|  | T333I | Destabilizing | 1.03 | -0.086 | -0.62 |
|  | T333R | Destabilizing | 1.49 | -0.093 | -0.02 |
|  | T343A | Destabilizing | 0.76 | 0.119 | -0.74 |
|  | T354M | Stabilizing | 0.66 | -0.245 | -0.31 |
|  | T379R | Destabilizing | 1.07 | -0.14 | 0.27 |
|  | T385A | Destabilizing | -0.78 | 0.019 | 0.11 |
|  | T385I | Destabilizing | -1.11 | -0.13 | -1.42 |
|  | T385P | Destabilizing | 1.15 | 0.088 | 0.14 |
|  | T413S | Destabilizing | 0.6 | -0.141 | -0.14 |
|  | T417I | Destabilizing | 0.52 | 0.059 | -0.3 |
|  | T417S | Destabilizing | 0.55 | 0.045 | 0.04 |
|  | T442I | Destabilizing | 0.05 | -0.377 | -0.09 |
|  | T450M | Stabilizing | 0.76 | -0.058 | -0.16 |
|  | T482I | Stabilizing | 0.56 | -0.137 | 0.24 |
|  | T510M | Destabilizing | 0.04 | -0.159 | 1.55 |
|  | T545I | Destabilizing | 0.58 | -0.012 | -0.45 |
|  | T545S | Destabilizing | 0.23 | 0.007 | -0.4 |
|  | T547N | Destabilizing | 0.69 | -0.332 | -0.18 |
|  | T547S | Destabilizing | 0.5 | -0.242 | -0.14 |
|  | T624M | Stabilizing | 0.42 | -0.375 | -0.17 |
|  | T627K | Destabilizing | 0.23 | -0.405 | 0.13 |
|  | T627M | Destabilizing | -0.13 | -0.353 | 0 |
|  | T681A | Destabilizing | 0.79 | 0.087 | 0.22 |
|  | T755I | Destabilizing | 0.38 | -0.253 | -0.34 |
|  | T762S | Destabilizing | 0.48 | 0.084 | -0.27 |
|  | T773N | Destabilizing | 0.66 | -0.289 | -0.45 |
|  | T95M | Destabilizing | 0.75 | -0.128 | -0.02 |
|  | V102M | Destabilizing | 1.37 | -0.008 | 0.06 |
|  | V124A | Highly Destabilizing | 2.34 | 0.332 | -0.6 |
|  | V124I | Destabilizing | 0.62 | -0.159 | -1.9 |
|  | V126I | Destabilizing | -0.24 | -0.26 | -0.99 |
|  | V126L | Destabilizing | 0.6 | -0.156 | -0.72 |
|  | V134G | Highly Destabilizing | 1.24 | 0.074 | -0.57 |
|  | V134L | Destabilizing | 0.84 | -0.076 | -2.76 |
|  | V134M | Destabilizing | 0.84 | -0.034 | -0.18 |
|  | V139G | Highly Destabilizing | 1.46 | 0.007 | -0.63 |
|  | V139L | Destabilizing | 0.52 | -0.19 | -2.7 |
|  | V139M | Destabilizing | 0.88 | -0.194 | -0.37 |
|  | V140M | Destabilizing | 0.92 | -0.006 | -0.35 |
|  | V187K | Destabilizing | 1.04 | -0.293 | -0.54 |
|  | V237M | Destabilizing | 0.26 | -0.072 | -0.64 |
|  | V357M | Stabilizing | 0.9 | -0.023 | -0.75 |
|  | V381A | Destabilizing | 0.42 | 0.068 | 0.37 |
|  | V381L | Destabilizing | 0.49 | -0.25 | -1.37 |
|  | V412I | Destabilizing | -0.02 | -0.131 | -0.24 |
|  | V421A | Highly Destabilizing | 1.23 | 0.264 | -0.5 |
|  | V421F | Destabilizing | 1.45 | 0.004 | -2.03 |
|  | V425L | Destabilizing | 0.35 | -0.135 | -1.31 |
|  | V496M | Destabilizing | 0.92 | -0.297 | -0.31 |
|  | V570G | Destabilizing | 0.5 | -0.026 | -0.46 |
|  | V570I | Destabilizing | -0.16 | -0.125 | 0.24 |
|  | V587L | Destabilizing | 1.39 | -0.193 | -0.53 |
|  | V587M | Destabilizing | 1.53 | -0.1 | -0.71 |
|  | V591F | Destabilizing | 0.84 | 0.087 | -0.85 |
|  | V671M | Destabilizing | 1.38 | -0.041 | -1.15 |
|  | V690I | Destabilizing | 0.19 | -0.08 | -0.67 |
|  | V725M | Destabilizing | 0.51 | -0.446 | -1.04 |
|  | W113L | Destabilizing | 0.18 | 0.107 | -0.41 |
|  | W113R | Destabilizing | 0.48 | 0.083 | -1.73 |
|  | W509G | Highly Destabilizing | 1.4 | 0.141 | -1.1 |
|  | W594C | Destabilizing | 2.05 | 0.335 | -2.89 |
|  | Y116C | Destabilizing | 2.15 | 0.129 | -1.8 |
|  | Y116S | Highly Destabilizing | 2.14 | 0.235 | -2.12 |
|  | Y129C | Destabilizing | 1.24 | 0.169 | -3.51 |
|  | Y129H | Destabilizing | 0.91 | 0.219 | 0.48 |
|  | Y284D | Highly Destabilizing | 2.79 | 0.516 | -0.92 |
|  | Y311C | Highly Destabilizing | 2.25 | 0.239 | -3.74 |
|  | Y311S | Highly Destabilizing | 2.37 | 0.464 | -2.21 |
|  | Y360C | Destabilizing | 1.79 | 0.215 | -3.52 |
|  | Y387C | Destabilizing | 0.67 | -0.091 | -2.26 |
|  | Y471C | Destabilizing | 1.46 | 0.178 | 0.44 |
|  | Y471F | Destabilizing | -0.81 | 0.099 | 0.39 |
|  | Y471H | Destabilizing | 1.29 | 0.211 | -1.02 |
|  | Y502D | Highly Destabilizing | 2.74 | 0.378 | 0.61 |
|  | Y554H | Destabilizing | 0.9 | 0.008 | -3.02 |
|  | Y583S | Highly Destabilizing | 1.82 | 0.353 | -1.23 |
|  | Y610H | Highly Destabilizing | 2.4 | 0.342 | -2.43 |
|  | Y622C | Destabilizing | 2.54 | 0.254 | -1.78 |
|  | Y716C | Highly Destabilizing | 2.17 | 0.05 | -0.2 |

**Table S3**: List of pathogenic mutations in Myosin-3 predicted through structure-based tools.

| **S. No.** | **Mutation** | **PhD-SNP** | **SNPs&GO** | **MutPred2** | **MutPred2 Inference** |
| --- | --- | --- | --- | --- | --- |
|  | A336T | Disease | Disease | 0.89 | Gain of Relative solvent accessibility (Pr = 0.25 \| P = 0.04); Altered Transmembrane protein (Pr = 0.22 \| P = 3.4e-03) |
|  | A584E | Disease | Disease | 0.932 | Altered Metal binding (Pr = 0.31 \| P = 4.9e-03); Altered Ordered interface (Pr = 0.27 \| P = 7.5e-03); Gain of Strand (Pr = 0.27 \| P = 0.03); Loss of Allosteric site at Y583 (Pr = 0.27 \| P = 8.9e-03); Gain of Relative solvent accessibility (Pr = 0.26 \| P = 0.03); Altered DNA binding (Pr = 0.23 \| P = 0.01); Altered Transmembrane protein (Pr = 0.16 \| P = 0.01); Loss of Catalytic site at Y583 (Pr = 0.16 \| P = 0.02) |
|  | C401R | Disease | Disease | 0.923 | Altered DNA binding (Pr = 0.40 \| P = 1.2e-04); Loss of Allosteric site at R404 (Pr = 0.23 \| P = 0.03); Gain of Acetylation at K398 (Pr = 0.22 \| P = 0.03) |
|  | D219G | Disease | Disease | 0.823 | Loss of Helix (Pr = 0.27 \| P = 0.04) |
|  | D449V | Disease | Disease | 0.78 | Altered Transmembrane protein (Pr = 0.33 \| P = 5.8e-05); Altered DNA binding (Pr = 0.17 \| P = 0.04) |
|  | D462G | Disease | Disease | 0.945 | Altered Transmembrane protein (Pr = 0.27 \| P = 5.9e-04) |
|  | D546G | Disease | Disease | 0.922 | Gain of Acetylation at K550 (Pr = 0.22 \| P = 0.03) |
|  | E281K | Disease | Disease | 0.847 | Altered Disordered interface (Pr = 0.28 \| P = 0.04); Altered Ordered interface (Pr = 0.25 \| P = 0.02); Altered Metal binding (Pr = 0.23 \| P = 0.04); Gain of Allosteric site at H285 (Pr = 0.19 \| P = 0.05); Altered DNA binding (Pr = 0.18 \| P = 0.03); Gain of Pyrrolidone carboxylic acid at Q277 (Pr = 0.04 \| P = 0.05) |
|  | E467A | Disease | Disease | 0.915 | Altered Ordered interface (Pr = 0.25 \| P = 0.03); Altered Transmembrane protein (Pr = 0.12 \| P = 0.03); Loss of Sulfation at Y471 (Pr = 0.02 \| P = 0.03) |
|  | E501K | Disease | Disease | 0.889 | Loss of SUMOylation at K503 (Pr = 0.19 \| P = 0.04) |
|  | F287V | Disease | Disease | 0.881 | Altered Coiled coil (Pr = 1.00 \| P = 1.5e-05); Altered Disordered interface (Pr = 0.34 \| P = 0.01); Altered Ordered interface (Pr = 0.25 \| P = 0.02); Altered Metal binding (Pr = 0.24 \| P = 0.04); Altered DNA binding (Pr = 0.19 \| P = 0.02); Gain of Allosteric site at Y288 (Pr = 0.19 \| P = 0.05); Altered Transmembrane protein (Pr = 0.11 \| P = 0.04) |
|  | F365C | Disease | Disease | 0.818 | Loss of Strand (Pr = 0.26 \| P = 0.04); Loss of Methylation at K366 (Pr = 0.10 \| P = 0.04) |
|  | F365S | Disease | Disease | 0.857 | Gain of Intrinsic disorder (Pr = 0.42 \| P = 6.4e-03); Altered Ordered interface (Pr = 0.28 \| P = 0.04); Gain of B-factor (Pr = 0.28 \| P = 8.3e-03); Gain of Phosphorylation at Y360 (Pr = 0.24 \| P = 0.04); Altered Stability (Pr = 0.14 \| P = 0.02); Gain of Methylation at K366 (Pr = 0.11 \| P = 0.03) |
|  | F532S | Disease | Neutral | 0.92 |  |
|  | F652S | Disease | Disease | 0.832 | Gain of Intrinsic disorder (Pr = 0.34 \| P = 0.02); Altered DNA binding (Pr = 0.21 \| P = 0.02); Altered Coiled coil (Pr = 0.14 \| P = 0.03); Altered Stability (Pr = 0.12 \| P = 0.03) |
|  | G182A | Disease | Disease | 0.893 | Altered Ordered interface (Pr = 0.27 \| P = 0.05); Altered DNA binding (Pr = 0.21 \| P = 0.02); Altered Transmembrane protein (Pr = 0.17 \| P = 0.01); Loss of Methylation at K185 (Pr = 0.16 \| P = 0.01) |
|  | G246D | Disease | Disease | 0.918 | Loss of Acetylation at K247 (Pr = 0.34 \| P = 2.4e-03); Gain of Strand (Pr = 0.26 \| P = 0.04); Altered DNA binding (Pr = 0.26 \| P = 7.9e-03); Altered Transmembrane protein (Pr = 0.25 \| P = 1.5e-03); Loss of N-linked glycosylation at N241 (Pr = 0.04 \| P = 0.03) |
|  | G769S | Disease | Disease | 0.928 | Gain of Relative solvent accessibility (Pr = 0.30 \| P = 8.3e-03); Altered DNA binding (Pr = 0.21 \| P = 0.02); Gain of Allosteric site at F765 (Pr = 0.21 \| P = 0.03); Loss of Acetylation at K767 (Pr = 0.19 \| P = 0.04); Loss of Catalytic site at K767 (Pr = 0.08 \| P = 0.05); Altered Coiled coil (Pr = 0.07 \| P = 0.05) |
|  | H154Y | Disease | Disease | 0.861 | Altered Ordered interface (Pr = 0.40 \| P = 9.6e-04); Loss of Intrinsic disorder (Pr = 0.37 \| P = 0.04); Altered Metal binding (Pr = 0.29 \| P = 0.01); Gain of Allosteric site at F156 (Pr = 0.24 \| P = 0.02); Gain of Pyrrolidone carboxylic acid at Q149 (Pr = 0.05 \| P = 0.04) |
|  | H285Y | Disease | Disease | 0.896 | Altered Metal binding (Pr = 0.33 \| P = 0.01); Altered Ordered interface (Pr = 0.33 \| P = 1.5e-03); Altered Disordered interface (Pr = 0.33 \| P = 0.01); Altered Coiled coil (Pr = 0.33 \| P = 0.01); Gain of Allosteric site at Y288 (Pr = 0.19 \| P = 0.04); Altered DNA binding (Pr = 0.18 \| P = 0.03); Altered Transmembrane protein (Pr = 0.11 \| P = 0.04) |
|  | H359Q | Disease | Disease | 0.888 | Loss of Allosteric site at M358 (Pr = 0.27 \| P = 8.7e-03); Gain of Phosphorylation at Y360 (Pr = 0.23 \| P = 0.05); Altered Metal binding (Pr = 0.18 \| P = 0.04) |
|  | H557Q | Disease | Disease | 0.738 | Altered DNA binding (Pr = 0.25 \| P = 7.5e-03); Loss of Acetylation at K560 (Pr = 0.24 \| P = 0.02) |
|  | I115F | Disease | Disease | 0.908 | Altered Ordered interface (Pr = 0.25 \| P = 0.02); Gain of Allosteric site at Y118 (Pr = 0.25 \| P = 0.01); Altered Transmembrane protein (Pr = 0.20 \| P = 5.3e-03) |
|  | I115S | Disease | Disease | 0.944 | Altered Ordered interface (Pr = 0.46 \| P = 1.3e-04); Gain of Allosteric site at Y118 (Pr = 0.29 \| P = 2.8e-03); Altered Transmembrane protein (Pr = 0.21 \| P = 4.2e-03); Altered Stability (Pr = 0.21 \| P = 0.01) |
|  | I158S | Disease | Disease | 0.901 | Altered Ordered interface (Pr = 0.35 \| P = 5.8e-03); Gain of Intrinsic disorder (Pr = 0.34 \| P = 0.02); Altered Metal binding (Pr = 0.23 \| P = 0.04); Gain of Allosteric site at F156 (Pr = 0.22 \| P = 0.03); Altered Transmembrane protein (Pr = 0.11 \| P = 0.03) |
|  | I158T | Disease | Disease | 0.777 | Altered Ordered interface (Pr = 0.34 \| P = 7.6e-03); Altered Metal binding (Pr = 0.24 \| P = 0.04); Gain of Allosteric site at F156 (Pr = 0.21 \| P = 0.03); Altered Transmembrane protein (Pr = 0.11 \| P = 0.03); Altered Stability (Pr = 0.11 \| P = 0.04) |
|  | I175F | Disease | Disease | 0.919 | Altered Transmembrane protein (Pr = 0.15 \| P = 0.02); Altered Stability (Pr = 0.15 \| P = 0.02); Loss of N-linked glycosylation at N172 (Pr = 0.01 \| P = 0.04) |
|  | I221T | Disease | Disease | 0.74 | Gain of Intrinsic disorder (Pr = 0.30 \| P = 0.05); Altered Stability (Pr = 0.11 \| P = 0.04) |
|  | I264T | Disease | Disease | 0.919 | Altered Stability (Pr = 0.27 \| P = 6.7e-03); Altered Ordered interface (Pr = 0.24 \| P = 0.03); Altered Transmembrane protein (Pr = 0.20 \| P = 5.3e-03) |
|  | I458T | Disease | Disease | 0.881 | Altered Transmembrane protein (Pr = 0.27 \| P = 8.2e-04); Altered DNA binding (Pr = 0.16 \| P = 0.04); Altered Stability (Pr = 0.15 \| P = 0.02) |
|  | I507T | Disease | Disease | 0.818 | Gain of Intrinsic disorder (Pr = 0.33 \| P = 0.03); Gain of B-factor (Pr = 0.25 \| P = 0.03); Altered Ordered interface (Pr = 0.23 \| P = 0.05); Loss of SUMOylation at K503 (Pr = 0.18 \| P = 0.05); Altered Transmembrane protein (Pr = 0.11 \| P = 0.03) |
|  | I522M | Disease | Disease | 0.785 | Altered Transmembrane protein (Pr = 0.23 \| P = 1.9e-03); Altered Metal binding (Pr = 0.14 \| P = 0.04) |
|  | I525N | Disease | Disease | 0.921 | Altered Transmembrane protein (Pr = 0.42 \| P = 0.0e+00); Gain of Allosteric site at E526 (Pr = 0.18 \| P = 0.05) |
|  | I674T | Disease | Disease | 0.772 | Loss of Loop (Pr = 0.28 \| P = 0.01); Loss of Strand (Pr = 0.27 \| P = 0.01); Gain of Allosteric site at R672 (Pr = 0.20 \| P = 0.04); Altered Stability (Pr = 0.11 \| P = 0.03); Loss of N-linked glycosylation at N677 (Pr = 0.04 \| P = 0.02) |
|  | K543M | Disease | Neutral | 0.849 |  |
|  | L103P | Disease | Disease | 0.956 | Altered Ordered interface (Pr = 0.32 \| P = 3.3e-03); Loss of Helix (Pr = 0.30 \| P = 5.2e-03); Altered Transmembrane protein (Pr = 0.22 \| P = 3.0e-03) |
|  | L106V | Disease | Disease | 0.755 | Altered Ordered interface (Pr = 0.24 \| P = 0.03); Altered Transmembrane protein (Pr = 0.22 \| P = 3.8e-03); Gain of Allosteric site at R109 (Pr = 0.21 \| P = 0.03) |
|  | L268P | Disease | Disease | 0.964 | Loss of Helix (Pr = 0.30 \| P = 5.2e-03); Altered Transmembrane protein (Pr = 0.24 \| P = 1.8e-03); Altered Ordered interface (Pr = 0.24 \| P = 0.04) |
|  | L291I | Neutral | Neutral | 0.261 | - |
|  | L298F | Neutral | Neutral | 0.627 | Gain of Strand (Pr = 0.28 \| P = 0.01); Altered Coiled coil (Pr = 0.19 \| P = 0.03); Altered Transmembrane protein (Pr = 0.16 \| P = 0.01) |
|  | L298P | Disease | Disease | 0.914 | Altered Coiled coil (Pr = 0.38 \| P = 0.01); Gain of B-factor (Pr = 0.29 \| P = 5.2e-03); Loss of Loop (Pr = 0.27 \| P = 0.02); Altered Transmembrane protein (Pr = 0.19 \| P = 7.1e-03); Altered Stability (Pr = 0.11 \| P = 0.03) |
|  | L302Q | Disease | Disease | 0.918 | Gain of Strand (Pr = 0.27 \| P = 0.02); Altered Coiled coil (Pr = 0.16 \| P = 0.03); Altered Transmembrane protein (Pr = 0.14 \| P = 0.02) |
|  | L388M | Disease | Neutral | 0.721 | Loss of Loop (Pr = 0.27 \| P = 0.04); Gain of Phosphorylation at Y387 (Pr = 0.23 \| P = 0.05); Gain of Methylation at K384 (Pr = 0.17 \| P = 9.5e-03); Loss of Ubiquitylation at K384 (Pr = 0.17 \| P = 0.02); Loss of N-linked glycosylation at N392 (Pr = 0.01 \| P = 0.04) |
|  | L400F | Disease | Disease | 0.647 | Gain of Strand (Pr = 0.27 \| P = 0.03); Altered DNA binding (Pr = 0.24 \| P = 0.01); Gain of Allosteric site at R404 (Pr = 0.23 \| P = 0.02) |
|  | L428F | Disease | Neutral | 0.822 | Altered Metal binding (Pr = 0.27 \| P = 8.3e-03); Altered Ordered interface (Pr = 0.25 \| P = 0.02); Altered Transmembrane protein (Pr = 0.20 \| P = 6.1e-03); Altered Stability (Pr = 0.11 \| P = 0.04) |
|  | L436F | Disease | Disease | 0.816 | Altered Ordered interface (Pr = 0.25 \| P = 0.02); Gain of Acetylation at K435 (Pr = 0.24 \| P = 0.02) |
|  | L617P | Disease | Disease | 0.789 | Altered Stability (Pr = 0.39 \| P = 3.1e-03); Loss of Helix (Pr = 0.28 \| P = 0.03); Altered Ordered interface (Pr = 0.27 \| P = 8.4e-03); Altered Coiled coil (Pr = 0.25 \| P = 0.02) |
|  | L621F | Disease | Disease | 0.642 | Altered Ordered interface (Pr = 0.27 \| P = 8.1e-03) |
|  | L621R | Disease | Disease | 0.903 | Altered Ordered interface (Pr = 0.26 \| P = 0.01) |
|  | L700P | Disease | Disease | 0.97 | Gain of Allosteric site at R704 (Pr = 0.26 \| P = 7.7e-03); Altered Metal binding (Pr = 0.23 \| P = 0.04); Loss of Catalytic site at N697 (Pr = 0.14 \| P = 0.02) |
|  | L771P | Disease | Disease | 0.951 | Gain of Relative solvent accessibility (Pr = 0.27 \| P = 0.02); Gain of Allosteric site at F766 (Pr = 0.20 \| P = 0.04); Gain of Acetylation at K767 (Pr = 0.19 \| P = 0.05); Altered Metal binding (Pr = 0.17 \| P = 0.03); Altered DNA binding (Pr = 0.17 \| P = 0.04); Gain of Catalytic site at K767 (Pr = 0.08 \| P = 0.05) |
|  | M166V | Disease | Disease | 0.861 | Altered Ordered interface (Pr = 0.27 \| P = 6.8e-03); Gain of Allosteric site at Y163 (Pr = 0.21 \| P = 0.03); Altered Transmembrane protein (Pr = 0.20 \| P = 5.3e-03) |
|  | N105S | Disease | Disease | 0.68 | Altered Ordered interface (Pr = 0.24 \| P = 0.03); Altered Transmembrane protein (Pr = 0.21 \| P = 3.8e-03); Loss of Allosteric site at R109 (Pr = 0.20 \| P = 0.04) |
|  | N225S | Disease | Disease | 0.792 |  |
|  | N241D | Disease | Disease | 0.858 | Altered DNA binding (Pr = 0.26 \| P = 7.8e-03); Altered Transmembrane protein (Pr = 0.19 \| P = 6.8e-03); Loss of N-linked glycosylation at N241 (Pr = 0.04 \| P = 0.03) |
|  | N483S | Disease | Disease | 0.72 | Altered Transmembrane protein (Pr = 0.15 \| P = 0.02); Gain of N-linked glycosylation at N480 (Pr = 0.07 \| P = 0.02) |
|  | N677S | Disease | Disease | 0.721 | Loss of Loop (Pr = 0.27 \| P = 0.04); Loss of N-linked glycosylation at N677 (Pr = 0.04 \| P = 0.02) |
|  | P153S | Disease | Disease | 0.75 | Altered Ordered interface (Pr = 0.27 \| P = 0.05); Gain of Allosteric site at H154 (Pr = 0.20 \| P = 0.04); Altered Metal binding (Pr = 0.18 \| P = 0.04); Loss of Pyrrolidone carboxylic acid at Q149 (Pr = 0.05 \| P = 0.04) |
|  | P528A | Disease | Disease | 0.826 | Gain of Helix (Pr = 0.29 \| P = 0.02); Altered Transmembrane protein (Pr = 0.11 \| P = 0.03) |
|  | P676A | Disease | Disease | 0.773 | Loss of Loop (Pr = 0.28 \| P = 0.01); Gain of Strand (Pr = 0.27 \| P = 0.02); Altered Transmembrane protein (Pr = 0.10 \| P = 0.05); Gain of N-linked glycosylation at N677 (Pr = 0.05 \| P = 0.02) |
|  | Q289E | Disease | Disease | 0.877 | Gain of Helix (Pr = 0.27 \| P = 0.03); Altered Ordered interface (Pr = 0.26 \| P = 0.01); Altered Metal binding (Pr = 0.23 \| P = 0.02); Altered DNA binding (Pr = 0.19 \| P = 0.02); Altered Coiled coil (Pr = 0.12 \| P = 0.03); Altered Transmembrane protein (Pr = 0.11 \| P = 0.03) |
|  | Q289H | Disease | Disease | 0.886 | Altered Coiled coil (Pr = 0.51 \| P = 6.9e-03); Altered Metal binding (Pr = 0.28 \| P = 6.8e-03); Altered Ordered interface (Pr = 0.25 \| P = 0.02); Altered DNA binding (Pr = 0.17 \| P = 0.03); Altered Transmembrane protein (Pr = 0.10 \| P = 0.04) |
|  | Q420R | Disease | Disease | 0.72 | Gain of Helix (Pr = 0.27 \| P = 0.05); Altered Metal binding (Pr = 0.26 \| P = 7.8e-03); Altered Transmembrane protein (Pr = 0.20 \| P = 4.7e-03); Loss of Pyrrolidone carboxylic acid at Q420 (Pr = 0.08 \| P = 0.02) |
|  | R109G | Disease | Disease | 0.916 | Altered Ordered interface (Pr = 0.33 \| P = 3.2e-03); Loss of Helix (Pr = 0.31 \| P = 4.8e-03); Loss of Allosteric site at R109 (Pr = 0.23 \| P = 0.02); Altered Transmembrane protein (Pr = 0.21 \| P = 3.6e-03) |
|  | R109H | Disease | Disease | 0.775 | Loss of Helix (Pr = 0.29 \| P = 0.02); Altered Ordered interface (Pr = 0.26 \| P = 1.0e-02); Altered Transmembrane protein (Pr = 0.22 \| P = 3.4e-03); Loss of Allosteric site at R109 (Pr = 0.21 \| P = 0.04) |
|  | R144G | Disease | Neutral | 0.86 | Loss of Helix (Pr = 0.30 \| P = 5.2e-03); Altered Ordered interface (Pr = 0.28 \| P = 5.8e-03); Gain of B-factor (Pr = 0.27 \| P = 0.01); Gain of Strand (Pr = 0.26 \| P = 0.04); Altered Transmembrane protein (Pr = 0.22 \| P = 3.1e-03); Altered DNA binding (Pr = 0.15 \| P = 0.05); Gain of Methylation at K147 (Pr = 0.12 \| P = 0.03); Gain of Pyrrolidone carboxylic acid at Q149 (Pr = 0.05 \| P = 0.03) |
|  | R148G | Disease | Disease | 0.78 | Altered Ordered interface (Pr = 0.27 \| P = 8.5e-03); Gain of B-factor (Pr = 0.26 \| P = 0.02); Altered Transmembrane protein (Pr = 0.19 \| P = 6.9e-03); Gain of Methylation at K147 (Pr = 0.12 \| P = 0.03); Loss of Pyrrolidone carboxylic acid at Q149 (Pr = 0.05 \| P = 0.04) |
|  | R238G | Disease | Disease | 0.879 | Altered DNA binding (Pr = 0.24 \| P = 7.8e-03); Altered Stability (Pr = 0.21 \| P = 0.01); Loss of ADP-ribosylation at R238 (Pr = 0.20 \| P = 0.04); Altered Transmembrane protein (Pr = 0.19 \| P = 6.7e-03); Gain of Ubiquitylation at K235 (Pr = 0.15 \| P = 0.04); Gain of N-linked glycosylation at N241 (Pr = 0.04 \| P = 0.02) |
|  | R244C | Disease | Disease | 0.869 | Altered DNA binding (Pr = 0.40 \| P = 3.6e-04); Altered Disordered interface (Pr = 0.32 \| P = 0.01); Loss of Acetylation at K247 (Pr = 0.30 \| P = 4.5e-03); Altered Transmembrane protein (Pr = 0.25 \| P = 1.5e-03); Gain of N-linked glycosylation at N241 (Pr = 0.04 \| P = 0.02) |
|  | R244G | Disease | Disease | 0.923 | Altered DNA binding (Pr = 0.43 \| P = 2.2e-04); Altered Disordered interface (Pr = 0.27 \| P = 0.05); Loss of Acetylation at K247 (Pr = 0.27 \| P = 7.4e-03); Altered Transmembrane protein (Pr = 0.25 \| P = 1.3e-03); Altered Stability (Pr = 0.16 \| P = 0.02); Gain of N-linked glycosylation at N241 (Pr = 0.04 \| P = 0.02) |
|  | R244H | Disease | Disease | 0.822 | Altered DNA binding (Pr = 0.33 \| P = 1.3e-03); Loss of Acetylation at K247 (Pr = 0.28 \| P = 5.7e-03); Altered Transmembrane protein (Pr = 0.24 \| P = 2.0e-03); Altered Stability (Pr = 0.10 \| P = 0.04); Gain of N-linked glycosylation at N241 (Pr = 0.04 \| P = 0.02) |
|  | R443C | Disease | Disease | 0.915 | Altered Ordered interface (Pr = 0.26 \| P = 0.01); Altered Transmembrane protein (Pr = 0.14 \| P = 0.02) |
|  | T333A | Disease | Neutral | 0.893 | Loss of Relative solvent accessibility (Pr = 0.29 \| P = 0.01); Altered Metal binding (Pr = 0.26 \| P = 5.4e-03); Altered Transmembrane protein (Pr = 0.24 \| P = 1.9e-03); Loss of Allosteric site at E329 (Pr = 0.20 \| P = 0.04) |
|  | V124A | Disease | Disease | 0.744 | Altered Ordered interface (Pr = 0.25 \| P = 0.02); Altered Transmembrane protein (Pr = 0.21 \| P = 4.7e-03) |
|  | V134G | Disease | Disease | 0.883 | Altered Ordered interface (Pr = 0.30 \| P = 0.02); Gain of Relative solvent accessibility (Pr = 0.26 \| P = 0.03); Altered Transmembrane protein (Pr = 0.25 \| P = 1.5e-03); Altered DNA binding (Pr = 0.23 \| P = 0.01); Gain of Allosteric site at Y135 (Pr = 0.19 \| P = 0.04) |
|  | V139G | Disease | Disease | 0.848 | Altered Stability (Pr = 0.37 \| P = 3.6e-03); Gain of Intrinsic disorder (Pr = 0.36 \| P = 0.01); Loss of Helix (Pr = 0.27 \| P = 0.04); Altered Transmembrane protein (Pr = 0.26 \| P = 9.5e-04); Altered Ordered interface (Pr = 0.26 \| P = 0.02); Gain of Relative solvent accessibility (Pr = 0.25 \| P = 0.04); Altered DNA binding (Pr = 0.24 \| P = 0.01); Gain of GPI-anchor amidation at N136 (Pr = 0.01 \| P = 0.02) |
|  | V421F | Disease | Disease | 0.872 | Altered Metal binding (Pr = 0.26 \| P = 8.6e-03); Gain of Strand (Pr = 0.26 \| P = 0.04); Altered Transmembrane protein (Pr = 0.25 \| P = 1.5e-03); Gain of Pyrrolidone carboxylic acid at Q420 (Pr = 0.08 \| P = 0.02) |
|  | V591F | Disease | Disease | 0.808 | Gain of Allosteric site at Y589 (Pr = 0.27 \| P = 5.9e-03); Altered Metal binding (Pr = 0.25 \| P = 0.01); Altered Ordered interface (Pr = 0.25 \| P = 0.02); Loss of Relative solvent accessibility (Pr = 0.25 \| P = 0.03); Altered DNA binding (Pr = 0.24 \| P = 0.01); Altered Transmembrane protein (Pr = 0.20 \| P = 5.8e-03); Gain of Catalytic site at W594 (Pr = 0.12 \| P = 0.03) |
|  | W509G | Disease | Disease | 0.919 | Altered Coiled coil (Pr = 0.99 \| P = 3.4e-05); Gain of Intrinsic disorder (Pr = 0.34 \| P = 0.02); Loss of Strand (Pr = 0.26 \| P = 0.04); Altered Ordered interface (Pr = 0.25 \| P = 0.02); Gain of Allosteric site at F514 (Pr = 0.21 \| P = 0.03); Altered Metal binding (Pr = 0.17 \| P = 0.03); Altered Stability (Pr = 0.16 \| P = 0.02); Altered Transmembrane protein (Pr = 0.13 \| P = 0.02) |
|  | W594C | Disease | Disease | 0.967 | Loss of Allosteric site at W594 (Pr = 0.37 \| P = 1.0e-03); Altered Ordered interface (Pr = 0.36 \| P = 3.4e-03); Loss of Relative solvent accessibility (Pr = 0.30 \| P = 0.01); Altered Metal binding (Pr = 0.24 \| P = 0.02); Altered DNA binding (Pr = 0.23 \| P = 0.01); Altered Transmembrane protein (Pr = 0.20 \| P = 5.0e-03); Gain of Disulfide linkage at W594 (Pr = 0.15 \| P = 0.03); Gain of Methylation at K597 (Pr = 0.14 \| P = 0.02); Gain of Catalytic site at Y589 (Pr = 0.13 \| P = 0.03) |
|  | Y116C | Disease | Disease | 0.921 | Altered Ordered interface (Pr = 0.47 \| P = 3.1e-04); Altered Transmembrane protein (Pr = 0.22 \| P = 2.9e-03) |
|  | Y116S | Disease | Disease | 0.936 | Altered Ordered interface (Pr = 0.49 \| P = 2.0e-04); Gain of Allosteric site at Y118 (Pr = 0.21 \| P = 0.03); Altered Transmembrane protein (Pr = 0.20 \| P = 5.7e-03) |
|  | Y129H | Disease | Disease | 0.911 | Altered Ordered interface (Pr = 0.38 \| P = 1.6e-03); Altered Transmembrane protein (Pr = 0.21 \| P = 4.3e-03) |
|  | Y284D | Disease | Disease | 0.931 | Altered Coiled coil (Pr = 1.00 \| P = 1.5e-05); Altered Metal binding (Pr = 0.45 \| P = 1.6e-03); Altered Ordered interface (Pr = 0.38 \| P = 1.4e-03); Altered Disordered interface (Pr = 0.34 \| P = 0.01); Gain of Helix (Pr = 0.27 \| P = 0.04); Altered DNA binding (Pr = 0.21 \| P = 0.02); Altered Transmembrane protein (Pr = 0.12 \| P = 0.03) |
|  | Y311C | Disease | Disease | 0.893 | Altered Metal binding (Pr = 0.35 \| P = 0.01); Altered Ordered interface (Pr = 0.34 \| P = 2.9e-03); Altered Transmembrane protein (Pr = 0.14 \| P = 0.02); Loss of Sulfation at Y309 (Pr = 0.02 \| P = 0.02) |
|  | Y311S | Disease | Disease | 0.892 | Altered Ordered interface (Pr = 0.34 \| P = 2.7e-03); Altered Metal binding (Pr = 0.21 \| P = 0.03); Altered Transmembrane protein (Pr = 0.14 \| P = 0.02); Loss of Sulfation at Y309 (Pr = 0.02 \| P = 0.02) |
|  | Y502D | Disease | Disease | 0.921 | Altered Coiled coil (Pr = 0.71 \| P = 3.3e-03); Gain of Intrinsic disorder (Pr = 0.38 \| P = 0.01); Gain of B-factor (Pr = 0.33 \| P = 5.3e-04); Altered Ordered interface (Pr = 0.31 \| P = 3.9e-03) |
|  | Y583S | Disease | Disease | 0.938 | Loss of Allosteric site at Y583 (Pr = 0.41 \| P = 5.2e-04); Altered Ordered interface (Pr = 0.38 \| P = 1.4e-03); Altered Metal binding (Pr = 0.31 \| P = 3.6e-03); Gain of Relative solvent accessibility (Pr = 0.28 \| P = 0.01); Loss of Catalytic site at Y583 (Pr = 0.17 \| P = 0.02); Altered Transmembrane protein (Pr = 0.14 \| P = 0.02); Altered Stability (Pr = 0.11 \| P = 0.04) |
|  | Y716C | Disease | Disease | 0.905 | Altered Disordered interface (Pr = 0.32 \| P = 0.02); Altered DNA binding (Pr = 0.28 \| P = 4.7e-03); Loss of Allosteric site at N712 (Pr = 0.28 \| P = 6.7e-03); Altered Ordered interface (Pr = 0.25 \| P = 0.02); Altered Transmembrane protein (Pr = 0.10 \| P = 0.04) |
